# Supplementary material for: A tyrannosauroid metatarsus from the Merchantville Formation of Delaware increases the diversity of non-tyrannosaurid tyrannosauroids on Appalachia
Source: PeerJ. 2017 Nov 30;5:e4123. doi: 10.7717/peerj.4123 (PMC5712462; doi:10.7717/peerj.4123)
Supplement: Supplemental Information 1 — Includes scorings for YPM VPPU.021795 for use in the matrices of Brusatte et al. (2014a) and Carr et al. (2017) and strict consensus trees generated from the analysis. [file peerj-05-4123-s001.pdf]

Supplementary Information for “A Tyrannosauroid Metatarsus from the Merchantville Formation of Delaware increases the diversity of non-Tyrannosaurid Tyrannosauroids on Appalachia”

Chase Brownstein

TNT v. 1.5 dataset data

Analysis within Tyrannosauroidea (matrix after Carr et al. 2017).

YPM_VPPU.021795

???
???
???
???
??????????????????????????????????1??10??0??

```
    ,— Allosaurus
    |          ,— Compsognathus
    |     ,-58|   ,— Ornithomimosauria
|—|     |     `-58—— Maniraptora
    |     |        ,— Guanlong
    |     |    ,——|    ,— Proceratosaurus
    `—100|    |    `——|    ,— Kileskus
          |    |        `——|   ,— Yutyrannus
          |    |            `—————— Sinotyrannus
          `52/0|   ,— Dilong
               |   |        ,— Aviatyrannis
               |   |   ,——|  ,— Eotyrannus
               `——|   |   `—|  ,— Stokesosaurus
                   |   |      `—0— Juratyrant
                   |   |      ,— YPM_VPPU.021795
                   `———|      |— Appalachiosaurus
                       |      |— Dryptosaurus
                       |      |  ,— Xiongguanlong
                       `————+————— Timurlengia
                              |   ,— Bistahieversor
                              |   |      ,— Qianzhousaurus
                              |   |   ,-91—— Alioramus
                              `——|   |  ,— Albertosaurus_libratus
                                  |   |-97— Albertosaurus_sarcophagus
                                  `——|    ,— Iren_Dabasu_taxon
                                      |    |— Teratophoneus
                                      |    |— Lythronax
                                      `——|   ,— Nanuqsaurus
                                          |   |            ,—— Daspletosaurus_horneri
                                          `—|        ,—92—— Daspletosaurus_torosus
                                              `——77|     ,— Zhuchengtyrannus
                                                     `—81|    ,—— Tyrannosaurus_rex
                                                          `—88—— Tyrannosaurus_bataar
```

Supplementary Figure 1. Strict consensus tree from 32 MPTs for the analysis of YPM VPPU. 021795 in the matrix of Carr et al. (2017). Numbers greater than 3 are bootstrap values (100 replicates in TNT, Goloboff & Catalano, 2016).

Analysis within Coelurosauria (TWiG matrix after Brusatte et al., 2014).

YPM_VPPU.021795

???
??????????????????
???
??????????????????
???
??????????????????
???
??????????????????
??1?????0???
0??
??????
???
??????????????????
???
???????10?1???????
??
0?0????????????????
?????????????

```
   ,--- Allosaurus_fragilis
   |     ,-- Sinraptor_dongi
   |     |    ,-- Bicentenaria
   |     |    |        ,-- Zuolong
|--|     |    |    ,------- Tugulusaurus
   |     |    |    |    ,-- Tanycolagreus
   |     |    |    |--75--- Coelurus_fragilis
   `--100|    |    |   ,--- YPM_VPPU.021795
         |    |    |   |         ,-- Kileskus
         |    |    |   |    ,--62|    ,-- Sinotyrannus
         |    |    |---|    |    `--63|   ,-- Proceratosaurus_bradleyi
         |    |    |   |    |         `---- Guanlong
         `--64|    |   `----|    ,-- Dilong_paradoxus
              |    |        |    |    ,-- Juratyrant
              |    |        `----|    |   ,-- Eotyrannus_lengi
              |    |             `----|   |    ,-- Xiongguanlong
              |    |                  `---|    |    ,-- Dryptosaurus
              |    |                      `----|    |    ,-- Appalachiosaurus
              |    |                           `--65|    |    ,-- Bistahieversor
              |    |                                `----|    |         ,-- Gorgosaurus_libratus
              |    |                                     `--65|    ,--96---Albertosaurus_sarcophagus
              |    |                                          `--69|    ,-- Alioramus
              `----|                                               `--66|    ,-- Teratophoneus
                   |                                                    `--74|    ,-- Daspletosaurus
                   |                                                         `--94|    ,-- Tarbosaurus_baatar
                   |                                                              `---97-- Tyrannosaurus_rex
                   |
                   v
         TO OTHER COELUROSAURS
```

Supplementary Figure 2. Strict consensus tree from 81 MPTs for the analysis of YPM VPPU. 021795 in the matrix of Brusatte et al. (2014). Numbers greater than 3 are bootstrap values (100 replicates in TNT, Goloboff & Catalano, 2016).
