# Supplementary material for: A tyrannosauroid metatarsus from the Merchantville Formation of Delaware increases the diversity of non-tyrannosaurid tyrannosauroids on Appalachia
Source: PeerJ. 2017 Nov 30;5:e4123. doi: 10.7717/peerj.4123 (PMC5712462; doi:10.7717/peerj.4123)
Supplement: Supplemental Information 2 [file peerj-05-4123-s002.tnt]

xread 853 155 ;

Allosaurus_fragilis

?10000?0000000100011001000101200111011001 0??

00000000000010000000000100010000000000010101001000000010010100000000000010000000000????

000000000000000?

100000000000100001001010000000100010000001100000010000000000000000000001000000000000?0?000?

0011000010000001000011100021011000000000200000000?0000??0000000?00000??000000001 0000[01]0?

0????????0000020?00?0000000101000?0?0010000?00000000000000100001 0?01000??0000000?000?

0000020000000000000?00000?00?0010000000020000?000100??001100000[01]0?

00000000100000000110000000000100011000000021121001001000100?1????000000000100??10?0000?

0000000000011000?100?000?0?1000000000????000?

000100100001010000000000000000010200000000000000000000010000000000020101[01]0000100000000000000000

0100000010?

00010000010201100000000001100000000100000000000000100100010000000000000000000000000000000000

00000000000??000000000000000000000?00000100000000000000?

Sinraptor_dongi

?10000?000?00010001000000001012000010110010?00?00000000000100000?00000100?10?

000000000101010010000000100101000000000001??????0??1?10?0?0??00??0??????000000000?010??

01001010000001?0?1000001100000010000000000000?000?000?00?000000?0?000?

00110000100100010000010000210 1???000000000000000000?0000??0000000?00000??000000001 0000???00?00??

00?????2????????????1010?????????????????????????????????????1??????0??000??

0000000020000000000000000000?00?00??0???00020000?0001?0??0011000?????????

000100000000000000000010000011000000021021000000?010?00?1000000000000000100??01?0000?00000000000?

11000?100?010?0?100?00?001000?0010000100100001010?0?000000000000112000000?00????00000010000?

000000020101??0??1000000????????????00001 0010?0000?00001020110101 0?00011000000??1?

000000000000001001000??0???000000000100?00000000000000???????00000000??0000000000000000000000000?

0010?00000?00000001

Dromaeosaurus_albertensis

?0??0010000000000010??0???0?01??01110????1111????101?1001100??

000000100111110000?0000101001??

1???11?1?0????00??????00?0?00??1?????????1?00??011000100?

0???????????????0???0000?00000??0000??0000000?00000??

00???

00???????????????????????????????????????0????0?????????010121?000??0???00?0?????????0????0??0??0???

0??????0?0100?00?00????????????????????????0????00000100000???????0???????0001001?0?0????00??00?????

0?0?10???????????000?1000000010????000000000001120100000000?0?

00010???0??000?0??????????????0??

0?010?????????????????????????0200000??0?1?0??110????????????????000?

0?

Deinonychus_antirrhopus

?0010????1???????1??0000?01110001110001111111100?1??00001110100000?0100111?1000?

0100101000?110001100?1121011????00110111121??????1?11011100100111000001002211101010202220?

01111211201?1111000000000010001101001000000000011000000?00010?0010002110000011110110000??

00000122000000000?0????000????0???????????00000??000000000000?[01]0?0?????????????20000?000?

000111000?0?0010000?000000000000000101?0???00000??0100000?000?00000000000000000000000000?

00100000000????????0?1????00111?????????0000?????00001?10?0010001?000001010??00?0???????00??0?0??

0?????00001000000??01?00?0?00000001????????????????????????0?0?1001000?000?00?????????????????

00000000001120100?0000?0?????00000000?00001000100?????00000?1101010000100??1?

0000??????????????????00000?000000000?00?100?00??0001?1??0?000010?100000000010?010??00?

0000000000000000000000000??0????000000000100??1101?000110???200?10001?0

Velociraptor_mongoliensis

?001001001000012011200001011100011100012111?10?

01010000111010000000100111110001010010100001100011001112101111100011011112111101011111111001001

110000010022111011102022020111122120111111?00000000011101101001000000000011000000000011?

0010102110000011100110000000000001120000000000000?000000?0000??0000000??0000??0000?00000000[01]0?

00??000?00000020000?000?000111000?0?001000???0000000000000010100???00000??0100000?

00000000000000000000000000000000?011000000010[12]21?0001101100011100000?100000000?0100001?10?

0010001?0?0001010100000???0??0?01??00?0????00000100000??01?0000?0000000100 1?0?0???000??

00110100?0???01000?000?000001001000?00000000000??00?112010000??????00000?????????????01001000?

000001110101?0?0?00?0100000110?01?01000000000000000000000000?000100000?000?101010?

00001001000000000010?0100200000000000000000000000000??00001000000000010000?1?10?

0011010020001000100

Balaur_bondoc

??

???1?2??1????0???10?????????0????111[01]11101001?1?00001???2???0111?202?02?0?1?22122???????????????

2110001001000??00??1???????????0?00??0?????????????????????1?????2?

00??00000000?[01]???0????????????20??????0???0111000?0?

001000???010?00?????010?00???21??0?00000020?100??0000??????00???0?000000000?00??0?00000????????

010????????1????????

00000???

???0100?0?0??0?

00?1101010000?00?01000?0110?01?????????????000?00??00?????00100000?00??1??????0000100100?0000?????

0?0???????????0000000000000000000??000????000000210????????000?10???200?1??????

Tsaagan_mangas

?00100100100001201120000101010001?1000121111100010110000100??000000100?

11110001010010100001100??1??????0?????????????????1????????11?

111??00??00??00?0000???????????

2110000011?00?0000000?00001????0000000????000000?0000??0000000?00000???

000??????????????????????????20000???0?0?0??

10???0????????0121?

00???0??00011?0????????????00?0100001?10?0010001?0?00010101000?00???0??0?01??00?0????

000001000000??0??0000?00000001001?000???000??001101?0?0???????????????0001001000000000000000??00?

112010000000????000000???00?00001??????????????00?110???

000?000?????????0?10?010?????10?????????????????02?0000000?????????????????????????????????????000?1?

1????110????0???000?0?

Bambiraptor_feinbergorum

?0010??001000012010[02]00?010111000?1100012111?1000101?00011?0?0?000?0100?1111000?
010010100??1110?1100?1??100?0?1100[01]1011??2????010101011110?001?100000111?211?0101?202202?
111022100???11100000000010001101?000000000001100000000011?0010?021100??11?110??0000?
000000122000?00000?0??00000??0000??0000000?1?000??000000000100??0??0?0?0?0?0000020000?00000?011?
000?0?0?1000??000000000?00010?????00000??0000000?000?000000000000000000000000?00?011?
100000000121?000110??00011101100?10000000010100001?10?0110001?0000010001000000???0??0??1??00?
0????000001000000??01?0000?00000001??1?0?????000??001101????????????????????0????????????????0?000??
00?1120100????????????0000000?000010001011000?000001110101000010?00100000110?01?010000000?
00000?000000000?0001?000??00001?10?0?100010?100????????????????0?000?????00??00????000000??
0000100000000100?0???10000110100200?10001??

Tianyuraptor_ostromi

??01??????????????????0????111??????????????0??1???????????????????????????????0?0?0?1010??0???0?????????
0?0?0?1??01?0112???????1??00?011110?001?1100001112?1??0?1102223?21111022?0???1?110??????????00?
11????000??0???1??00?0??01???1011?0???0??????1?0??????????0????2?000?
0???????????????????????????????????????0??????????0?00?00????00000?0?00?000?0?
0???00?1?0????10??????????????0?0????????20?
101?????????????1???????????0000?10??000??1??????????????????????????
0???
??????????0??????????????????????????????0???????????????????????????01?1?1???00??0?0????
000????????????????0?????0?0???00????0????0?0???0???????0???0?????0??0??????????????????0?????0???
0??????????????????????????0010????????0?0????????????0?0???

Sinornithosaurus_millenii

0001??????0??????????00???1110????1000111100?1????1??0????????00?00100??1????010100??100????????1??
1???????0???00?1??1???????01?1?11011110?0?????0000?00?201?01?112023?2?111022?2?1???1?????????
110001?1100?0?00010001??0000???011011110020000001111 00?00?00???000???20000?000?????0??000????
0????00000??1000??00?00?0?????0?0??0???????00?0?[12]0000???0?000100?00?0??010?0??

00?????????????????????0000??0000?0000??0?0?00?????000??0?0000?00?0???0111?11000????????01??

10000111?1100?1000000?01010000000000?0000000?0?0000000001000200???0??0??1??00?0????000000000000??00?

0000?00000001??1????0??000??001101?????????0???????????????????????????001000?010112010000???????

100?0???????????????????????????0011101????00?000?????????0?01???????????????0?0???00000??0??00000?

000?1??0?0?????10?????0?00?????0?0???0100??????0????00000????001?000?????0??000100?011?????0110????

0???000???

Microraptor_zhaoianus

0???????????????????100??0??010?0?1????0?01000000?????001?1??01?

2100?01???0110?1212111?01?101111111010??111000011012111??0112023?2?1110221201?11111??00?

0001110111110?100??000?11?000????1010111100?0?0???1?110???0?00??0?00???20??0?

000?????????????????????????????0?0???????00001000?[01]0?00?0000?001000200???0?000?01???10?0???10100?

000??1?0?000?????????000???????0?00?0000000000????000?00??000000000??1211111??00?021???011011???1?

1?1100?1000000??????????????????????????????

0??

?????????????00???1??????????????????????00??????????????00001????????01???1?10000?000???0000??0?01??

10?????0?00000?0???00????0????0?00??0????1????0000???10??0?00???0?010???????0???0?00??000??0?

000000?000?10?000000010??????????0?1??0?????1???1??

Graciliraptor_lujiatunensis

???020???

1???????????????????????????0???11?12?????????????????01011?1100001???????????????????????????????????????

0??0?010?[01]11?1??0??0??00??????????????0????????????????????00???????????????

200???0??????10?0???

000??0????????????10?0000??00?000??

10??????????????????????????????????

000???

??

0???????????????????????????????????1????00?00??????????????????????????????0000????00??????100??0??0???????

0?????????00?0?00?????01???????????????????0???00?????????????????0?000?10????????0?0??????????????????

Hesperonychus_elizabethae

??

??21??010?????????????221211?????????????????????

1????????????1??????????0??

1???

??

?????????????

0???

??

??????????????????????????????????01000?0110?01??????????????????????????????????0???0?????

1??000???

Pyroraptor_olympius

??

0????????1????????????????????????001??01??

1?????????0?????????????0??????

0???

???????????????????????????????????????010??0??

0???

??

???????????????????????????0?0??????????0???0???????????????

0?????????????????

0??

Rahonavis_ostromi

???0???????????????

011121???1?01?011112?12???????????0??11??011??????0?01111?10111120?3?2012101?121??21110001000?

011101101?0100????0??1?????0??

10001011111??

0011000[01]0???????????????2????????????1100?????????????????????????010100???????????????000000?

000000000100000000?00000000?001??01???0????????0?1?11?????1????????

00??

???100?1??0?0??

0011??????0?????0?100000????01??10???0?0????00?0???00???????????00??0?00?1????0000??0?0???????0???

010???????????000?????????0000000?000?10??00?0001????????00?0?11?0??0?????????

Buitreraptor_gonzalozorum

?0010????????????????00001011?00??????????100?10??01?0?????????00??001??????????0210??100?

0010111100111?110???100011012[012]121???????0110111010?1?1??0??????11?101??12[02]23??01?1?1?120???

11?0??0??1?011?0?1110000?????0??1????????1101000110011000???0??????0000??0?00?10200?00?0??????????

0???????0?000101???0?????00000000000[01]0??????????01000100?0?0?00??0?10000?0?001100??

0000010000000001?100????????????????000????00???0?00??????????0?00?000?0010?00??????????????1????0010??

1100?0000???00?0????????1?11?00?0???010?0101?0??0????00000??1100????????0000???0?0??0??

0??????????????100?????000??00?0?????????????????????0????????????????0?000???0?1?2????????????????

000???????0001001000??0??00000111010100???0?001??000???????01000???00??0?????????0?0?000?????????

00????0?1000100?0???????01000?????0?00010000000000????000000??0??01000?0??001???????10????11100?

00010001??

Neuquenraptor_Unenlagia

???

11211111111???1????1????????????0??1001???????????01111?101111202202011 1?121202011?10000??0?010??

111100000????0??11????0??110110?110??????????????????????0?????

220??0?00000?0??????????????????1????????????1100?0?0?

0011000?00000????????????0???????????????00000?00000000000000000000?00000000?

00??

??

???????????????????????11011?0?????0011???01???????00100000110?01?010000000?00?0100???00?????????

000?00?00?1????10????01???????00???010?????????????00???0????000000??000010??0?000010?????????0??

111001000???????

Austroraptor

?0000????????????????????2?1?0????????12?0001?????????????????????0?011???????????210??10?????0011?

001????11????????????????????????????????00????????0??????????????????????????????????????1??????0?0?0?0??

[12]1??????????0????0????????????????11?00??01??00??????????????1[12]20??0?

0???????????????????????????????????????0??00?00???????????????????????????????????????0?0?001100???00??

0000000???

?00?0??????????0?10100?0000010??1???????1??????????????????????????????0??01?01???????????????????0?

10?00????????????00000?000?00????????????????0??0010000?????????????????000???????

0001001101??????????????101??????????????????????????????000???????00?00????????????0?????0?

0???????????????????????????0?0001000?????0???????0000???????00??????0?????01?1????1?????1?0??0001??

Shanag_ashile

??????????????????1??1010110????????????????????????????????00??011??????????

01001011???

???????????????????????????0????0????????????????21100001???00??????????????????00??

0??

???

????????1?10???00?0?

000000??

?????????????????0??00??00???????????????????

10??0?0???????????????????

0?????????????????????????????00???1????????????????00????

Mahakala_omnogovae

??????????????101?????????????????????1?00???????0?0011??????????????????????02????1???00??0?11?011?

211??1?100011012210????????????????00??1110000?100?211101?2??????????????????0111100????0?

011001101?00000??00??11?0?????0000?????00????????0????????????0????1[01]2?0??????0?????????????????

0??1?00???????????0000010000[01]0????????????????????????????10??[01]0???0?0000???0?????????????

100????0??0???000000??0??001??0???000000000?00000000?001??00?000????1???0??????011?0????????

000000???????????????????0???0???0?????

000?00????????0???????????????00010????????????????????0???????????????????0000?????0?01?000???????

000??????????00????00100000???????0100000?0?000?100?0?000?0????0?0?0???0????1?10???00???????????

0????010?20????0?0??0???????000000000??0??0100000000010?0????1?000???10????01????0?

Atrociraptor_marshalli

???????????????????[02]00?0??1010??????????????????????????????????00??010????????0?

0000101001??

????????????????????????????0????0????????????????21?00??0??111?????????1???????????

0??????????????????????????????0????

0??

??00001?1????1???0?0?000?

01???

????????????0?????0?00???????????????????

0???0?0?????????????0??????

0????????????????????????????000??0??1??????????????????00????

Utahraptor

???????????????????[02]00?0??????????0?001?????????????????????????????????????0????0101??1????0?1100?

1??1????????0??011?????????????101?1???????????0?????????0?01????????????11?????0111?00??0?

001000100???10????????11??0???????0?????0???????????1???0???????0???10???0???

0??00000???????????????????20000?

000000??00???????0??000000000000?0000?????0?

10??00?????000001?????????????????????????1???0??00010?

0??

???01?????????0?

0?????????????0?????0????????????????00????????????0??????????????????????????????0?

0???????????????????1?????????????????

Adasaurus_mongoliensis

?0010?????0????2???????????????0?????0?1??11?1?0?101??001??0?????0????0?1???0?????????????01100?11?

0111?10?111[01]100??011?1?1??????1?111111??????????????1022111010102?2202?1?11221001?111000000?000?

11010010010??00??00110?0?0?0001??00100?????????11????????00?0????1??????????0?????????00??00???000?

0???????????000?0010000[01]0????????????????200?0???00??

01110???00?00000000000000000000000000?002??11?00???00???

21?000??0??0001?1?????????0???0??0??????????????????????????????????0?????????1??00??????0000010??

000??0??00?0?0?00????????0??????????0?????????????????????????0??????????????????0???????

11201??100000??0?01???????????????0?0???00?

0????????000?00????10?????????????????????????00??????0??00???????????000000??000010000??

00010??????????01????????????????

Achillobator_giganticus

?????????????????????????01?1??0000101??????0?01100?

11210???????0??011?11??????????101????????????00??10220??11010102102?011011?001?21110?0????00?000?

101????0???????1???0????001??00100021100??0??100????????

0??0000????????????????????20000?0?0?0?0??

10??00???0?00000??000???????0?0000????????0???????????????

0??????????0???????????00???????????00?001???0?0?000??

1???

??10???????????????0100??????????????????????????100000??0101?

0100000?1000??????????0?????????0??01?????1??0??0???????????????01??0?????????0??00?????????0000000???

0000100???????1????????????????1?1?????0?00??

Saurornitholestes_langstoni

??111?????????????11????0?????????????????100101?00?

11000110011121011011100?1011?1?1?????????????111?????????000111221?1?1?1?2?0???????????????????11??

0????00??001101??00???00?0?1??00?0000010?001000????????1110011??????0????1220???????

0????????????????????0000???????????0????????????????????????????????0???00??

1??0??????????????????????????????????????0???00??????????

0??????00111????????00?00????????????00?01??0?0?0?000?

00??000??00?1???0?

000????????????????????????????????0??0000000????????????????????0????????????????1?

0??0??????0????????????????0???????????????

1??01?????????????0?????????00???????????????????????????????11??????????????????0????

Saurornithoides_mongoliensis

?00??1?1??1101???0?110001?1000????????2?????????????????1?010?100?0010??1????

0001110101?????????1???0??1?????100??1???02012020?010??

10[01]1?11110????????????0???10?01??????0??1?0???????????010??11000??0?1?01?0?????0?000??????0??

0000????0?1000?0??0???????????0000???0????00100?

0??00????????0?0????

00000000000000?20?021000000000000201001?10???1110?1????????????00?0100000??2??11?0?0?1?

0100010100??00???0??0?????0??0?????????????00?????????????????????????????????????0???????1????????

000????????????????0????????????????0???????00000?????????????0????????0?

0????????????????????????????1?010???????????00????00000???????00??00????0?0???00???????????1??????

0??000?0?????0???????????????????0?00???????0?101?0????????1???0??????0?0?00

Zanabazar_junior

?00101?12?110100?001?000??100000????2022000?21??0??11100?????100?001???1????

000111010100?????????????????1?1000?1020?1???

2????????????????????????????011????2???????????00??00???11????????0??110000000100000????1??

00???????00?000?????001??0?00?????????????0000??0?00????????

0[01]0??

00010000??000??2????1??0???00000201001?11???1110?1????????????00??100000??2??11?0?0?1?0100010100?

000???0??0?????0??0?????????10??000??00?0??????????????????????000?00011010??????????????????000?

00000????0000???000?001???????????0????00000???????????????????0??????????????????????????????????

0?????????????0?0????????000?????????????0?????00???0?????????????????01?00?

0000??????????????????????????????????0?00????100100???????????1????000?0?

Xixiasaurus

???????????????????01000101110??????2?22???????????????????0?00??001????????000201??

01?0??00?

1??0???00????????????????????1?000000???01?

0000????00???????000000???00????????????????????????

0???0??????

0???0?0?????????????????????????????????00????110100??2?01100?

0?1?0100010100000???0??0???000??

0??010?000???????????????

00000??000??????0??????

0????0?0??????????????0????????????0100??????????????0???????????????????????1?010??????1??????????

000???

Byronosaurus_jaffei

?????101???101?1100110001011?0??????20220??????????1?100??????0000001??11????000211??01?0?

0???????010121????????0??02???1????0?0??

0???????21??????????0????0????1???????????11000000?1?00????0??0?000??0???000000???????????

0??????????????0?0????0????

0010??

000?????????????????????????????000200011??0????????1????????????00??100000??2?011?0?0?1?

0100010100??00???0??0?????0??0?????????????00???????????????????????????0???????????

0??????????????????00?000000????????0??00??00?????????0???????0?0000??????????

1???000?????????????0????

000????????????????????????00?0100?????????????????????????????00?????????1?0?0?1????1??????????000?0?

Sinornithoides_youngi

?0??01?????????????1?000??1??0????00???2???????1??????????????00??0010???????0001110?01??????11??

001???????????0011?102121011???1?00?1?101?0???100000????21??01??02??30???11?0??001??1110?????0???1?

000110001?0??0?0?1??00?0???0???0010?0??00000????0??0??0???00???0?2000000?0???????????????????

00?????1?00??000??????????[01]0?0????????0??0?20??0?00000?0??1000???0?1000???00?0?????????0??010?

0000???000000??00??0??000????000000000000000020?0210000000????????101??????1?010100??1??

000??????????????????????????????????????0???

000??0??0?0?00?????????0???????????????

00?110101?000?000???????????????1??????0?????000???00?0???0???0?00??0??1????0?????100????0?0????????

0???000?000???00??10?000?????0??00???00000000010????0?1?0?0101????0????0????

Mei_long

?0????????0????????11010?????0?11?002012100001?1010??100??????00010010??1?0000?021???01?

001101110001 1?2110?01???0110222111?1?????111?11100?0?1?1000001?012111?1??1200302?1110221?[01]?

0111100?0101001000??110011?0?0000???00000??000100?1??0?1?000000?001?0?0000?000001020000?

000?????????00?0??0??00??0101????????0?0000010000[01]0?0????????0100020?00?00000?0111000?0?

0000000?0000000?000?01?1?0???00000????00000?000000010000??00000000000?000021?01100000??0???20???

1010??101100?1100?10?0000?110??0??0??????????0?1?01????0?????00???????????????0?????????????

00??????????????????1???0??????0????0????0????????????????????????????????????0?000?????????????

0???????0?0000?????????0?????????????????????1?1?0?????????????????????????????0??????00???00?00?0??????

0????0????1?0?000?1001??00?0001?0?010???0???0?0000000000?000????00???0???00?000000100?????????

01??????????0?0???

Jinfengopteryx_elegans

?0?????????????????1?01???2000????????1??00?0????1???100??????00?00??0??1??0?000?1???0???

1??????????????????????02????2?200???????1101110??????100000?????1??????????????????2??0??????????

0?????????????????0?0??00???00??????????????01?000001???0??0?0??????0????200?0?

000???????????????????????????0??????0?000???0?0??0?0??????????????00?0???0?0?01???

0???10?0000???????????0??

11?01??????????????000?110?????0?01??1????0?????????0?????00?????????????????????????????

0??????????????????????????????0????0????0????????????????????????????????????0??

00??????????????????????????0?????????????0????????0????1??0????00????0??????????????????????

0???????????????0?0??00??????00????1?0???0????????0?????????????????000??????????????0?0???????

00?????????001?0???????????????????????000???

Anchiornis_huxleyi

000100??????????????11010??11100?????0?2?000??1???11???????????000??01??1?????00020???01??0?101???00?

0??010?01???01101220??01?????011111110000??100000010?21?10011020?202?1?1122121???1?1?000??1?01?

000121100010?1000?1??00?0???????0?11?011000001???0??00?????000???030000?000??????????0??????0????

0??????0??????00000?0000[01]0?0????????0000020000?0?00??0?10000?????10?0???0????????????????10?

01000??0?00?00??00?00000????????????00000?0?00??0100000000????????00??1110?1?1?1100010??000?110??

0??0?01?010?0?0?1??101?00??0??00????????????????0?????????1???000??0???2??????????1?????????00????

0????0???????????????????????????????????0?000??01?????????0???????0?0000?????????0???000??????

000??????1???00??0000100000??0?01?????????0?????0?0???00?00?0??100?00??00???11?0?0000???????

0??????????0????1000????0????0??0?0000000???00?0???????00100???0???0?01??????????000???

Xiaotingia

?00???????????????????1?1???111000????0?2??????????11????????????001001??1???00??020???01??0??0???1?

0?????10?01?????1??????10??????01?01111?10???1000001011?1110?1102122?????102?12[01]??????????

10?????????1?010001010000?1??0?10??00???0010?011000??1???00??0?????000???22000??

000??????????????????????0000?????????0??000?10000???0????????010002????????????11?000?0?00???0???0?0?

0????0????????????0100??1?00000???0?0000???????????????????0??0?02????10000????????00??1??????1?1100?

10??000?110??0??0?01??1????0?????0???0?????0???????????????0?????????1???

00?????????????????????????????0????0????0????????????????????????????????????0??00???1?????????

0???????????00?????????0???????????????0011??????0???000010?000???????????????0???????0???00000?0??

100?00??00?1??1?0???????0????0??????????0?????000?????0???0??000?000?????0??????????0?100???0?????

0101????0???000???

Aurornis

?0010???????????????0101???11100???000?1?0???????????????????????00?001????????0?020????1??0????????

0????????0????0??0?221?00??????01?01111??0???10000??001?011????02122020??10??????????????????????

0??100000?000000?1??0??0??00????111?011?0000????0???000???0000???20000?

000?????????????????????????????0??????000??0???000?0??????????00???2??0????????0?0?010????????

0???????????????????10?0??00??0?00?00?0000000???????????????0???0??????1??010000????????0???1?1?1??

1??1000100?000?110??0??0?01??10???0?1??101?001???000???????????????0?????????10??000??0??02???????

00???????????00????0???0???0???1?????????0??????????

00????????????????????????0???01???1???00??0000100000???????????????0???????0???00??0?0??100?00??

00????1?0???0????????0??????????0?????00??????0??????0?000000??????????????00100???????????0?????????

00????

Eosinopteryx

00010???????????????1?01??????000????0?1?0?????0??1??????????????00?001????????00020????1??0????????

0??????????????0??0?231?10???????1101111??0???100000?001?1?1??1?02002?20??10??????????????????????

00?1??0001000000?1??0??0??00????011?0???00??????00?0?00???0000???20000?

0000???????????????????????????0??????0000?0?10000?0?0????????00???2??0????0???0?1?010????????

0???????????????????????0??00??0?00?00?000?000????????????????0?????00??01???000??????????0???11??1??

1??1??0??0?000?11???0??0????????????????????0?????00???????????????0?????????10??

0????????????????????????????????0????0???0???0???1?????????

0???????????0???????????????000??????0??0011??1???0???000?100000???????????????0?????0?0???00??0?0??

100??0??0??1????0?0?0????????0??????????0????100??????0??????00000000??????????????00100??????????0?

01????0????0????

Troodon_formosus

???1?1112?1101000001???0?011?0??????20220000210?0??01100????0?10??001?????????0111010100???

1111100101211111?1000?1020??11?????????????1?010?????000010??????????0?0?2?20?11001?0??01111000???

00010000021??01????000??1?0??010???1??0??0?1?00000?0?0???????00???????????????????????001??0?000?????

0??1?1?????????

00???

?????????????????????????0?????00000201001??0???1110?1?????????

0000???0?????

0000??00110?????????????????????0000001000??000000??0000000????????????????????00???????0?0??

00??????????????????????0??????????????????????????????????00000?000??0????????00??0???0??0????00??01??

0000?0?1??01?01?00?0????????????0?0??????????????????0?000?10?0?10?0?0?0???????????????0??0?

Sinovenator_changii

?0???0002?00001111001010 1?1110?011?02??2??0011???1?10100????0?000?001??????000001111 0?1?????11?

10100012110?001000110222?1?????????110111???????100?0???0?211?010112[02]0302?1110221201?11110000??

10011000111??110?000000???00001100010001100 11000??1??011??0000?00000??0??00000000000?000000?00?

0??0000010100[12]00??00000?0010000?????????????????20000??00?0101?

10???00?1000000100000000000000?0?00?020?0110?

00000000020?010?10??1011000???????000000?????0000?02?011??00?1?0100010?????

0??0????????0?00????1???????????????????0????????

0???00000??00???0????????????????????00?????????????00????0??????00111??????????????0000?0??0?01???????

1??0????0?0?0???0?0???????????0?0?1?1??0?????100????????0????0??01?01?0?0000?00??????0?00??0???0000?

0000000001?1???0?????0??1??1?????000?0?

EK_troodontid_IGM_100/44

?????0012??0???????????????1??11???????????1??????

0??1?

00000??01?1?001???00??????0?0??1?????????

0???1?

1??

??0?????????

000??

0???
??
0??????????1????0????????????0000????????????????????0?1?????????????????????????????
IGM_100/1126
?0????002?0000?11[12]01?010??2000?011?020121000110?01010100??0??000000000?11?000000211??00?
0??????????????????0????01102??????????????????????0??1000001???211?010?020020200010131012??
111000????0??2?001210001?00000011?00?0?020000?00100?1?00000111?01?0?00?000003??????000000?????
00??00?00?0??0??0000?00?00???0?0?????????0???
0?00???0000???0100000?00??0???0?00?00?????00?1?000?20?02????0000001020?111??0??101?001???????0?
000???
??
??
???
IGM_100/1323
?00???????0???11???1?000??20000?1??020121?00?1??0?0??????00?0?000?0????1??0000021???
0000???????????????????????011??????????????????????????????????00??1??????020?20200[12]102?
1011???????00???0????0??2???01??00??0????0?0???00?0?00100?1?000000?1?01?0?00?00?003??????
0000000????00?000?00?0?????0000?0?000??0?
00??00?????00?00?
0?00?????????1000??2???????????????????????????
01??
??
??
??
Archaeopteryx_lithographica
100?0000??000??112010010??1110?011000012100?10?0000??100111?0?000001000002?00000200??00100?1?
1???00?0??1?0?0???0021012311000????1011111111000?110000000112111010?1200302012102??2?2?111100000?
0001000000030?0000000001100000??00?0001100111000010 01?01000000???0000?030000?0000000?00110??00?
0??000?00??000???00?000000000[01]000?0????????00000?0000?00000001000000?0011000?000??000?

0000100000??00000?000000000000?0?00000000?00?0?[01]1000000?00???00000100001020?00001011101?
0101100?1000000110?00000?01?01?0000?0?0100?10100?000???0000?01??00?0????00???10??000?????????
000000?0?0????????000??0011000?????0??????????000000011??????00000?000000000??00???0???0???0?
0100???????000?0000????????0?0011101?10000?000?100000??0?01?01??????0????00?0???00?00?00?100000?
000?1?1000?0?0010010??000000?0?010020?1000?000000??0?000000?0000?000?????0?0000100001001???
0111????00?100010?
Confuciusornis_sanctus
10010??????????????1?000?00??0001???0??2??0??0??00??01???????000010000?10?0001?1?????????0?????????
1021?0?2???0?2????4??111?11010??13111000?11110000001121?1?1??12000?2?111023?2??12??10??11??
120211010030?00000000001102000??00?0101??11?100011001??????0?????000?123001[01]00120?????????10??
00??011200??01[12]0111110000001100001001000120000?00?0000?0??0??010000000?000211??
0010000000010102101011[01]?10?00000010100000000001010100100011000011000?0000000[01]000???
2000001101110?00??1100?10?0000?110?00000??1?00?0000?0?01?0?10100?00??????????1??00?0????0000000??
000??01?02???00100000?0????????000??00???0?????????????????????????????????????00010???0?0??00??
10????????????0???????0001?000???0????0?00111?0?1?000?100??????0??0??1?????????0????00?0???00?00?0??
100000??0??1??0?????0?????0??00???????010???????0?????0?0000?0?0??????0??0??????0?0000100?0?0?????
01????????????1????
Jeholornis_prima
10????0????????1???0???0?????001??????????????????0???????????0000?1???00?00?1?1?????????0????????
0????1?0?11???02111031??01?0???011031110001??1000001011?1??0??01?0???2???1?221?10???????01????
01011010030000000000??0??1000??00?010????1???000?1?????0???0??0??0??12300?10010??????????????????
0????00?00000??00?000000100000?0??001???0000?00000?0?1000120001000?0010000?000?0000?
000010000101100000?000000000000?00000?0100000000010000001000?00000001100????????011?1110???1?1100?
10?0000?110?00000??????????0?0???????0???????????????????????0???????????0??
00??????????????????????????????000??00??0??00???
0??????????????????????????????0?0??0000???????0?0011101?1?0???000???0000??0?01?????????0????00?0???
00?0??0??100000?00??1?10???0?00???????00???????010???????0?????0???0??0?000?00???000?????0?
0000100???0?????0??????????1?1?1??
Jixiangornis_orientalis

100?????????????????0010?????001????0?0??00000??????0100??????000001????0?0??1??0????????00?0???1?

00????1??20???02??1031??11?1?00011031110001101000001111210101021200002010 1?221?10?2?11?001??1?

01[12]1101003000?00?000?0??1000??00?0100??111?001?1?????????00?000000?12300?

00010??????????????????????0?????000??00?0000?11?0?10?11?0?????0000000000?001001121001 0?0?0000000?

000???0??0????0??010?00100??0000?001000000?00??0000??00000100000?00?0000?001100????????00??11????

01?1100?10?0000?11??00000??????????0?0???????0?????????????????????0??????????0??

0???????????????????????????????????0???00010???

0??????????????????????0????????????????????????????????0?1?00??00???????????????????????0?????0?0???00?

0??0??100?00??0?????0??????????????00?????????0?????????????????0??0?0????????????????????00100???

0?????0????????????1????

Yanornis_martini

?00?????????????????1?010?????0?11???0??2??0??????00???????????0000011???0????000?1???00??0????1???

0????????4?1???????04??????110?011031110?011?130010????????????????????????23?010?2??10??1??0?

0121[23]0000300000??0001???00?0???????0???21?1?0???????????????????0?????300100012?????????10???????

1?????????????????0?0011?00???0210?[12]??10100001010?1?11?112100111??0101?0101?10000?0?????

021[01]101311[01]1100[01]100?0?1?0??0?00??????0??00?1110[01]?1?00??1?0??01100????????01??1????????

1100?11??001?11??????0??????????0?0???????0?????0????????????????0??????????0??

0??0???0???

0??????????????????????0???????0?0??0?0??????????00111?0?1?00??100???0000??0?01?????????0?????0?0???

00?0??0??100000??00?1??0??????????????00?????????0?????000?????0????????000?00???????????????

00100?????????0??1?????????0?1??

Apsaravis_ukhaana

??????????????????????0???????????????????????????????????????0002?1?0??0????????????????1??11??10200??

1???5?100021??24??????1?0101103110000111130 03??000?1???1?201000?2?021?23?03?12?010?????1??

12130100?00000??20???1?20?0?????0?00??21????????????????????????????300?10?????????????????????0?

1[01]?????1?0????????20010?[01]001??21??1????????00000?0?1000121011111?

01011010[12]0111110000101021110?[23]120100?11??20?1[01]0010?11?110?1012112111111?0000?1?0??

01101????????011??????0?1????????

0001??

??0?????????01???0?????0??0?

0011100010001?1?0?1?0000????01?010??????????0000??000????????????0??0??1????????00?00100?????0??????

0???????0????0000000????00000????000????0?000?10????????0?0?11????0?????????

Yixianornis

100?????2?021??1?????0?01????0?1?1?0????????????????0100??????000?010???0?00?00120???10?01???1???

0000??1?0?40??0???0024???11?110001103 1110?001013000010 0021?101?21200302000102 3?23?0211?0??1??????

2130100100000??000?0??1000?20??0?000001???????????0??0??0???0??0?1230010001???????01??1????????

11[01]?????1?0?????10?20011000?11021??[12]??1010000101 0?111101121 00111100?0110???01?000???0?

01021[01]10?31201100[01]100?00100?00?002?101??????11110[01]?1?00??1?0?001100??????00011?11?01????

0100?10??000??1???

0????????????????????????????????0??

0?????????????????????0?????????????00?0????????00111?0???000?100?1??000????01?????????0?????0?0???

00????0??100?00??0??1????????0???0????00?????0???0???????0?????0???0????000?00????00??????0??

00100?????????0?11????0??1??????

Sapeornis

?001???????????????0?010??11000????00??21?0?00????0??????0????0000010??10?

00000020??????00????1???000????0?211??0210004??100?????2110111101001110000010 1121??00?212000?

201[01]00221210????1??01??1001210?10030000000000????2000??0??0100??0111?000???1???00??0???0000??

23000000000??????????????????????????0?00???0000000010000100????????0000020000?00000?010001000?0?

01010?0010000?0???010?1[01]10?11100??0[01]00?0000?0000?00?000??00?00??000001?00??0?

00001000????????001?1?1011?1?110001000000??1?000000??????????0?0???????010??00?????????01??00?

0?????????00??0??????????0?????000??0????????000??00???00000???

0?????0???0???????0?0100???????000??000?0???????0?00111?1?1?000?000???0000??0?01?0???????0??????0?0???

00?0??0??100000?000?1?10?0?0?0??0?1???000000?0???0????1??0???0?0??????0?000?000??000?????

000000100?0?0?1???00?1??????????1?1??

Neuquenornis_volans

???????12?????????20?????????????????????????00?????00???

00??????????????????????1?11011?1031110?01111000?????????????????????????????????????010???????????

[01]010030??0????0??????0?00???????????1???????????????????0???????

0200???0110???????21?????10110??0100?001?001?0??[12]??1???

01?0???0?????[01]?????????2???20?1??01????????????????10?1?????????1?0???1011???????0?000???

200000110??????????????????0??

0???00???

0????????????????????????000??0100???

0111?????0?????????????????????????????????0?0???00???????????00??0??1???0????????????????????????

2???????????????0??????????????????????????0010?0????????0?????????????????

Patagopteryx_deferrariisi

?0??

0010???2??0?

1???????????211101?????????????????????2?010001??100121301001?000???????1????????00?0?????

1??????????????????????0?????0??????????????00?1??????00??111110000?????????010000000????????0????????

000????0101?1210[01]00???[01]?10?0???01000001[01]0001?[12]00???30???????00?2000000?010120????0?

000[12]11001100000?[01]?0??0?100?1?200??01?0???0?001????????00???0?

1???0??????0?0??0?

110????????????????????0??????????????????????????0020000??00??????????0?0?00??0001?00000????????

00111?00100???????1?0000???????0100??0?0???0?000???00????0??????00??0??1??????0?10???????????00???

010???????00000000??0?????000000???00?1???00000010??????1???0????0???0?1???10?

Cathayornis

?0??????????????????1?010?????0?1??????????0???????????????????00?????????????00001???????0????????

[02]????????3?????2????4???0??11?02?1031110?011?1300?0?01111?0?00?12000?201?1022?23?????????1???0??

[12]1[01]1?0?3000?0???00????0??1??0??0100???111?????????0??0???????0????2300[01]0?

012????????????????????????????0??????0????0111??0010?20??1??10110?101????010?01200010010110[01]?

01020111110?[01]?1?????210120?11?01?0101011?0?000001???1?0?0?1[01]1?0???????????0??01110????????

01????????01??????????0???110?00000??????????0?0?0100?10100?000???????????????0??????????0??

00??????????????????????????????000??00?0?0????????????????????????????????????0??00??

00??????????????????0?0100???????0?????????????????00111?0???0?????????0000??????????????0?????0?0???

00?0??0??????00??00?1?1??0????????????????????????0????100??????0???0?????00?00???????????????

00100?????????0???????????000???

Concornis

??

00????????????????????0??11?02?1031110?0?1???0030????????????120?102??11?23??3?0???1???1???0?

1[02]11??0?3000?0???0????????????????001??1???????????????????????????

2300??0111????10?20??1?0101100101?0?0010001?0??[01]001?

110??0102?1???10???1[01]1?[12]??????????????[01]????????????00?????0?100??1?00??1?[01]1??0??????
110????????????????????1100110??

01???

???0??????????

00111?0?1?0????????????????????????????0?????0?0???00???????100?00??0??1???????????????????????????

0?????????????0????????0?????????????????0??0010??????????0????????????????

Gobipteryx

?0??????????????????1?01010?0?0?????????????????????????????11?000?0??????????1?

1???

2??11???????????????????????????????????

1????0??????????????????0????????1100100?00110?10?????00?????0???0??0???1?????????????10??????????11??

10000?0?100012000[12]001?1?0110???0?????????????1?121??[012]0?????1?0??1????????000?0???10?001111000?

1?11?0??00000??????????0?1?01????0???0?

0???0?????????????????

0??????????????????0?01000?000??00???

000???

0??0???0???

0??????????????????0????

Vorona

???

??12?010011101001112110???

00????????????????????0?????

2??

???1010111100001?

000000?????????????????????????????????????

00??

??

?????????????????????????????1???????00000000?00??0?

10001?????????????????????????????????????110?00000010??????????0????0?????1???1??

Songlingornis

?????????????????????0?0???00?????????????000?0????

0???????????????????????????????????????11??[01]???

3???0????0?????0?????

00??????????????????????????????0???????0?????????0?01????????????????????????????????????0???????????????

21??[12]?000100?010???1?

11011??0??

0?????????????1??1?????????????????????????????

0???

??????????????????????????????0??00??00????????????????0?0?0????????????????????????????1?1?????

0???1???????????????????????????????????

00??????????????????????????????????????0?1?????????????????????????0????

Pengornis_houi

?0010???????????????10010??0?00??????0?12??0??????0?????0??????000?001????????0?0?00??00???01??????

20???0???2??????????4????1?????2?1031111?0??????03??001?1????????????????????2??2??????????1??????211???

0?000??0??000???0??0??00???0???11?1?000??????????00????00001230000?000????????????????????????????

0?????0???0011??00100????????01[01]00?00??????0??1??012111[01]0?011010001[01][01]1?????1??1???10[12]

[12]0??1??1?11??????0????00?0????0??0[01]?1?00??1?11??0?0??0?100????????01?????00?01?1001011???1??

110?00000????0??0??0?0?0100??0?????00?????????????????0??????????0??000??????2??????????????????????0?0??

0???0?0?0???0?0??00???0???????0?0100??0?0??0??1?0????????????

00111?0?1000????0???0000????????????????0?????0?0???00?0??0??????00??00?1??0?0???0????1??????????????

0????1?00?????????0??????0?0?????00?1???????00100??????????1?1????????000???

Hesperornis

?0?????????????????00010100?00??????00021?001????00???00????1?000?001???1?0001?021???00??10101110?

2100??11?50100?210?24??11??10??00103111??????????3??0202100?1??01000???021023?03?12???0001??11?

1213110010??0?00??01?1?0000?000???00??2??1?1111????0???????10000??10?1?10?1121200110?1010??

1110011100??00?0???1010121011000012?0?000100100?0???10?0???00011??0?????????0???0????

0?????????????????????????20?11000011121102111100211111221220 02?0?????00??????0001?0???0?000?0100?

11???1??01??00000??????????0?1?0100??0?????00????????0???0??0??????????0??00????????????????00??

0????????0?0??0????0??????????????????????????????????????0?000???0????????????????????000???????0?0??

000???????0???01?????????????201??000????01?????????0????00?0???0??00?0??????00?????1??0?0?0?0???

0???????????????0?????000?????????0?????0000?????00?????????001?0??????0???1??????????000???

Baptornis

???

2?????????5?????????24??

212?????????????????????????????????????2???????????????????????????????????[12]????????????????????11001??

00??1??????????1210110000 12????012?0?????01?10?0???000????0?????????0???0??????????01?

[01]1[01]???????????????20?11000011121102111100211111?21020?

2??

???

?????????0???????0?0??000???????0???01???????0?????201??000????01?????????0????0000???0????????????

00?????1??????0?0???0???????????????0???????0?????????0?????0000?????00?????????0210???????

0????????????????????

Ichthyornis

?????00????????1220?????????????????????000111????0???????????000??1?????1000??0210??10???1?01111?

2100?1???5?000?21??34???01?100?001031110?011113003??0102?1??10212000????21023??3?12??10011??11?

1213110???00?0??20?????00????0??00????21???????????0?????????????012300[12]0?11???????????10[01]1?

111011101001020???101?1200111?101[12]?211022010100001010?

1111011211011110010110111010000101[01]1011211112312011001 1?12001[01]0010?1121102111100211 11[12]2?

100002?0??011?100?20???0110??1??00?00100?11??01??01???????????????????????????0?00?

00??1?0?????00?????????????????????????????0????

0100?????0?00?0000?00?????????000?0???????00??000??0?01?0000??????0??00111?00100?0?10201??000????

01?0???????0????0000?0?0???0?0??1???00??0??1??????000??00100?0???001??01??2?0?00000?00000000????

0000?????00?111?0?00001????10?1?0?0?11?0??0????0??0?

Iaceornis_marshii

???

????????????????????1101?010311??????113?03????????????????????????????????2??1??1???????

21?????????????20???1??

0??

21?02[23]11???1101010?11110112101???????????????????????????????11131401110111020010??1?1?

12110212110021??0100?11???

1??

???

00111???????0?10???????????????0??0??0??????0???????????????1??????????1???????????00????0???00???

01??????????????0??????1??????????????1???0?00????????????????11?0??0?????????

Limenavis_patagonica

???

??????????????????????????????????011?13??

20???

???10000[01]010101011?11[12]313011?

011??

???

???0?

0??

0000??

Lithornis

100????????????????01010100?00?111??2?02?00?0???????????????10000?00????02?101?1?????????1????????

2????????[67]?1????????????[12]??11000010311110011?13003??000210101?20200102??2102?????12?01000???

11?12130100?????0??200?01?2010???0?0?0???21???12???????????00???00000??300?11212100?

00011111010110?11[01]1101?1111?01?10121012?0111[12]1211122110100?

0101101111011210111100110001[12]11100001010101011111113130110[01]11102[01]0120110?

112110201100021111222100 10[01]?000011?1????????011?111??00???????????01???

1???

??0?????????????????0???0????????0?

0?????????????????????????????????1??000????????????????????????????????

0??0???????????????

0000???

Hongshanornis_longicresta

101?????????????????11010?????0?11???0????00???????????????????000?001??00????1?1?10???0??0??01??1?

0????????[234]??????????4???1??11?01110311100011113002 0?0112???01?20????????2??23?23??2??100?1??????

21[23]01000000000?000?01?1????????0?0????1?1?0?????????????0????0000?1?300???

012????????????????????????????00??????1?????0?????110????????01?0??11???0?10???21000111???110???2????

0????????????1??2000??000100?0??0?0??000????????1???1100??[01]?00??2?0???1???????????????????????

0100?11??001?11??????0???????????0?????????0??????0???????????????0??????????0??

0???????????????????????????????????0???0?0?0???0?????????

0????????????0???????0??????????????????01?1?0?000?0?10??????????????????????0?????0?0???00????0??

100?00??0?????0???????????1???00?????????0?????????????????0??0?0?????????0?????????00100?????????

0????????????1????

Liaoningornis_longidigitris

??

????????????????????????????3??1??0???100?1????0?

21211003000?????0?????????????????????????????????????

0???011?01??????????0???

0?????????????????????????????0?????01???2??????????????????????????01?1?0??001111110201000?

00??

??

??0????????????????????????????0?????0?0???00???????????00??

0?????????????????????????????????0???0010??????????

0??????????????1??

Crypturellus_undulatus

10????012?020002?2001010100?00?111?12002100001011100010???1?10000?000??102?001?1????????

1110111122100?01107?110??12?24??121?11010010311011011113003??001210001?20220112002102 3?23?12?

0100010011012130 1?0???0100020000 1?20000200?0?001021???120??01??????0????

00000103002112121[01]0100011111010110011011111111100101012101011211212111 12?

1010002101101011111210011111011000120110000111010101111103140101111 1020?120110?

11211020211002111122210010110??011?10102000001111110000??1100?10??01??

11??

0??

0???2??????????????????????

0???

0?????????????0??????????????????????????????

Gallus_gallus

100???102?021002?2000010100?00?111?120021?0001?1000001????1?10010000000000021101?1????????

101[01]1111221?0?01007?120??12?24??1[12]1?110120103110200111113003??001211111?2?2001120021023?23?

12?0100011011??213010010001000200100?20100200?0?0???21???121??01???0??00???

0000010300110212121111122110111111111111011000110101112101011201112111121101000210111111111112100

1111101100012011000010111010111111314010012110 21?121111?112110202110021111322200111?00?

0110001020?000111111?000??0010110??01??

11??

0??

0???2??????????????????????

0???

0?????????????0??????????????????????????????

Crax_pauxi

1001011?2?021000??001010100?00?111?12002100001110000 0100??1?1000000000000021101?1????????

1000111122100?111?7?120??12?24??121?11002010311121011113003??0002100?1?2?2001120021023?03?12?

0100011011?12130100100010002001?1?20100200?0?0???21???121??01??????00???

0000010300210212121111122110111111111111011000110101112101011201112111121101000210111111111112100

1111101100012011000010111010111111314010112110 21?111111?112110202110021111322200110100001 1000?

020?0001111110000?11100011??01??

11??

0???

0??2??????????????????????

0???

0?????????????0??????????????????????????????

Anas_platyrhynchus

100???102?021002?2101001100?00?111?10002??00?1?100010?0???1?10200000100?021101?1?????????101?

1111221?0?011?7?11???10?2410121?1101111031111011113003??0012000?1?2?2001120021023?13?12?

0100011011?121311?0???010002101?1?20100?00?0?0?1021???121?????????00???

00000113002102121111111221102111211111101100011110111210101020111211113120100101010?

1[01]111112100011100110001201100001011101111111231401010111021?111110?

111110102110021111[23]2210010210?0011?10?020?000111111?000??0100?11??01??

11??

0???

0??2??????????????????????

0???

0?????????????0??????????????????????????????

Chauna_torquata

000??1012?0210002?101010100?00?111?100021?0001010001010O??1?100000000??0021101?1?????????

1011101112100?111?7?11???1012412111?1000001031111011113103??0012101?1?2?200102?021023?23?12?

0100011011??213010010000000200101?20100000?0?0?1021???121??01??????00???

00000123001102121[12]11110221101111211111101100011110111210121020111211114010100101011111101112

110211100110001201100001011101011111231401011111021?111111?

1121102021200211112222001001000011000102000101111110000?11101?11??01??

11??

0???

0??2??????????????????????

0??

?0??????????????????????????????

Pedopenna

??

???0?0?0??

0010???

??

?????????????00?000?????0??0????00????????0???11???????????????

0???

??

??????????????????????????????????????00????0???00????????????00??0?????????????????????????????????

0???0?10????????????????????????????

Epidendrosaurus

???0??1????????????????10??????????01?????0???????0??????

1??????????????????????????????????001??1??0??????001??????????????????????????????????????0????????

0000030?0?0???0?????1???????????????1????????????????????????????3?

0???0???????0???????????????????0??0?0??????1???0?????????0???

0???0????????????????0??????000??????????????????????????100?00000??0?0??0?010????????00??????

1???????????0??

00???0????

0???

0011??????0????????????????????????????0?????0?0??00?????????0?00??0???????????????????????????????

0?????????????0????????0????????????????????0010??????????0?????????????????

Epidexipteryx

?01???????????????????1??????00?????0??2??0011?????????????????100?0?0????00001020???

10000???????????????0?[01]????1?1?123??10??0????0101?010?0??????00?1???????????022?????011?03?

0??????????0????011?0??0?10?000???00??1?1?0???00?0?00???0?0?????????1??2?????0???????2?0?????

0??????????0???????????????????????000?0????00??0?0??????????????0??0?0??????1???0?????????????00?

[01]0???10?0?0?0????10?00???0?????????1??11??

1????????????0?00?100?????0??????????0?????????0?????

0??0????

0???00200??02?????????0???????0?1????????????????????????????

0011??1???0?????00??????????01?????????0?????0?0?????????0????0?0????0????????0??????????????????

0???????00??????0???0????0?????????0??????????001?00?????????????????????0????

Incisivosaurus_gauthieri

?00?00?01?001??1??10001110100100 1?010?12000011000101011010111121010000100 1?1?000210??00?

0??

??????????????00??00??01?0??0?0?????????10000000100010 2?00010?100?????00000000000100001??0000???

0000101?1100??

0000??

???????????????????????????????????01020?00???0??000?0?0????????????11?1100000????1000000?0?0000?

000?0?000???1000??1??00?0????000?110??010??01?0000?00000???00?00????0100??0111000???????????????

00100000000010000????00200??02?1?000000000?????

00110???100??????????????0??

0?001?????????????????????????0201100???0?0?0??????0??????????000?

0?

Citipati_osmolskae

?001001001001??221000101111?01011?00010210001100001000100011012112 0?0100010111?1?????????

10111011001012110 01??201??002200111?01112001101001 00?1100000100021100 1??02012020??10111111?

2101000000000?00000000000000000000110200000000?0?001000?000002011???0???

[01]0000200012200010000000001102?1??0000??000001000101 0??0000020011000[01]0?00?

001000010102 0??????0????111000?0?0010000?000000000000?00100?0??0?0???0000000????00000?

2000000000000000000002?000?00000000002 0?000110??00[01]10?0110110000000011?1100001????1000001?0?

0001?11000?001???1002??0???0?0????000?000??000??01?0000?00000000000100?????100??

0111000????????????????10100000000100000????10201???2?1?0000000???

0???????????????????????????????????????10???00?00????????????????????????????0?0?0???00100????000?

00??0???0?00?????????0?0?0?00?0???0?00211??????????0??00?000?0???????0??????0?0000100?0100????

10?????????1?00?0?

Oviraptor_philoceratops

?00?0??????01?1????0??1??111?1011???0???1?0?11???01?0???0?11?121120?01?00?01?1?

1??????????????????????????????0???????????????12??1?????00???10??00?00??1??

0????????????????????????????0????0????0??0?????00?00000???2?00?0?????????????10000?0?0???????0??0???

0????00???0?00????????10?0?00??0??00?00?1?1????00???00?100????0????????01010?0?00?0?0????1?100??????

1100???0????????00?????????000?0??00000?????????????????????????????????????0??000?????20????1?0???

02?0??1101100???0011?1?????1????10????1???0?01????????01???????????????0?????0???00??0?0??0??00?????

00????????????????????0???1020????2?1?

0000000???1???1???0???

100????000???????????0????????????????0??0???????211?????????00??????0??0???????????????????????00??

0??????0???????????00???

Microvenator_celer

??21?20?0?????????????????????011?0?

11000121100???0?1?1002?????????????00?000?11000?????0010002???0000?????????????1110??0101100?

0000010???????0??0???00??11?20?0??00???0?1??0??????????????????????0???0?

2200000??0?0001????????????????????20000?000?01?????00?0??010?

00?000000000000010000?????00????????0???0??000002000000000000????????????10??0??????????????

1????????0???????????

00???

???0???00??0001?

01001??????????????010000?0?????100???????01?01??0000????00??????????????0?????0????????1????000?10?

000???0010?0001????1??????00??00?10??00000000??000?0??00000??????????1?01??11100??001??????

Caudipteryx_zoui

00010???????????????0?111??10?0001?10???21000???0?0?????????????21120?0????????0?1??0?????00????0??

00????1???0???01?????30?????0???0??0???01?0???100000?000?1??????0201202???1????101?11?????0????

01000??1?00?0?000000?1102000??00?0?001000100000?00????0???00?00000?1[01]2000000000???????????

0??????0000????1000???0000?00?0000?0?0??????????????20??0?0?000?11??00???????0?0???00?00????????????

1??0000???0000000?000000000??0?0?00????0000000??0??2000000??0????????01101100??01?110???0??000?

10???0000?????0????0?0?0001?00?????0????????????????0????00???00??0?0??0??0?????????000?0????????

1????0???00201???2?????????0?????????0??

0???????????????????????????1???????00??000???0000???????????????0?00??0?0???001?0?0???00?0???0????
10?????0???000??0?00?0???0?0???11??0????00????0?000?0000???0?0?????0?0?00100???0????????????????1?
10???
Ingenia_yanshani
?00?0?????????????????1?1?????1??????????????????????????????????21120?01000?0111?
1???????????????????????????1???01????2?00???1011120011??00000??100000100021??????02012020011011?
11101101?0000?00011?000000000??0000??11?2000?000?0?001000??????????????????0002?0??2200?1000000??
1?????0?0??0??0000?00??1?10???000??0??1??0[01]0??02??????0101??0??0???0?0?0101000?????1100???00?
0?????????????????00????0?00?00?000?0?000??0?0?000?0?0?0??00?0????00?000000????????0110??00?1?0?
1101000??000?
1???
??0011000?
001?0?01??101000??0101??11??0??????00000???00?????1?000000010??1?1?0??????10???0???0??0?000????
11???0???0100000001000000000??000100?000000010??????????0??1?0??00?1???1??
Rinchenia_mongoliensis
?00?0????0??????????0111?1???1?11?00010??00???0000?????00??1?12112??01000??111?1????????????????
1??????????????0?????220?????????2??1???00?0???1??0001000?1?????1???????????????1?????????0????0??0?
0???????0??00??001??2?00???????????????0??????00????????00??210??????2??0120????????10?0?10??0???
0?????????????0??
00???0?00??002?0?????????????00?1?1?00001????1??0001?
0?0001?11??0??01???1????????????0????000?000??000??01?00???0?000000?0????????100??0????
0????????????????????????????????
0??1?
1001????????????????????????????????0????????????????????0?????????????????0????????????
11??????????????????????0????????????????????????0???????????0?????????????0???
Conchoraptor_gracilis
?0010??????????1???00111?1???1?11?000??21000110?00????1?0????121120?010?0?0111?1??????????
01010110010??1?012?110??10????01?1?????20011000010???????00100021000101020020 20??101101[01]1?
11010000??00010000010?000??000?001102?0?0000?0?0010001000002001???????0?0?0200012??????

000000000????????10???0100100????????000??0?

010???00?00??

000002000000000000?0000?002?01?????????0?020?000??0??002?00???????????0011?1?00001????1??00?1?0?0?

01?11??0??01????0?2????????0????0?0??00??0?0??01?00???0?00?000?0?0??????100??0???

00????????????????????10???0???????

0??0?

0??????????????????????????????0?0????010?????010?????0?????0???????10???????00?0???????

21??????????????????0?000000???????????????0?1?00????????00?????????????0?0?

Chirostenotes_pergracilis

?????1??01?01101??0???1?110?0??????????????????????01010??????21120?00000201???1?????????????????1101?

12????1?12???0?????????????????101?1??????????00?100021?00101022120201110?111[01]??01??100???00?

100000200000??000?0?1??20?0?000?0?00100?????????????????????000?????[12]????10????????001????

00?????????????01?10???0????0011??00?????????????????20000?

00000???00000?00??00000?00?0????????000000?0???

0000?????001020?00???0?????????1????????0?001????????????????????1?0?00???

0???

1000001000???????00200???2?1?000??0000??????????0???????0?0??00???1000?0??????????????0??0??

101000?????1?0100?0??0?00??00000?001?0??1?1?000?01?????1?0??????10?????000??0??????02?1???0110?

1???????000000000???00????00??00010?0??0?????1?0??0????????0?1??

Avimimus_portentosus

?00?0???10011?00??0??1?1????????1???1?????00?11??00100110??????2?1???00?0??0111????????????

01101011010 1?1?00??100?????????????????????????0100??????????00?211?01??0201202010?011?0?1000100?

0???00011110020???00??0?0?01?02?000000?0?001000????????0?????????00?01??0?02001????????????????????

0??0???00??0???????????02001000??????????????????????????????0?????0?0?0010000?000000000????????????

2???????????[02]0?000?0000020?010000000000000??00?01??0??????0?????????????????????????????????1?

0???100??0?

1100?????????????????????0000001?0?00???????????????????????????????????????0???????

0001000001??????????????00001???????101001??0101?0100?0?000??001000??00???????0??0?1001??101????

0000100000?????1001101102?????101010000000????00?000???0010000000000010?0????1??01????0??00?1???

1??

Falcarius

??01?11100001111010?????1????00??????????00??????0000000??????100?000??0???????001010001?1??

000111010121010001200010 0??0000??????000001000100010?0000100?

0110010102203010101001000100000000000000110000000000000??000?10?00?0?000?0?001000????????0?

001???00?0?0?0??11?0??000???????000??00???0??00?00100?0?00??0000??0010000[01]0??????????0100020000?

00000001110?1?0?0010000?0010000000000010000???0100???0000000?000000000200000000000?00000000?

0000??00000?0?200000??0???00000?1100?00000000???????????????????000?00?00100????

0?????????????????????????????????0?????0???????????????01?00????00?00???????????????????????

0000001010010????0??00000001???0???????????????0?0???????0001000001000??1000??

1010000000100000000000?0101?010000000001?0000001000?0??00101000010?1?01?11?

1000100100000000011101002?0?10111100000010100010 0000??000001000?000000?0?10?10000?011011002?00?

10?

Beipiaosaurus

0??1???10?10?????????????1001001?100????

1??????????????000??0220?????????0000??0????0?1000000?0000??001???20220??0??0????0??00?0??00??????

0000????0???0??00111???0?0??0????0010?0???????????0??????????0???0??????

0??????????????????????????????????????00????001000?????????????00?00????????????????????????????????

10000????????????1??0100???000000???0??0??0?????0???????0000000000???0?0?00000?????????????10?????1?

1100??0??000?1?0?????0????????????????????

0??

?????????????0??0?00???????????????????????0???????0???????????????????????1???0?00??00???00?1????????

10?????0?0?????????00???????1?1?0?????????1??1?????10???000?0?00????????????????????0??0?01000?01010???

0?11??10??0?1?0???1?????????????????????1????

Segnosaurus_galbinensis

??21??10?0000?000???0100?001????????

1??????????1?0?0???????????????????0?10?1?00000????0???2001110010202022 1??0110201101?11001?00?00?

11000000021?0??00?01?1??0?0???00?0?010?00??????????0?????????00???????

0???????????????????????????????????0?0????0??????????????????????????????0??????0????1????1???0010000?

00100000??????????????????????????00?00000??10??????00?????000000000??21?0????00??????????????????

2??????????

0??

???

10?????????????0?00???????21020010?0101??02????????????0?00???????????????0??01??0??1??1????????2???????

0??110?????????1???????10100??1?1112221011111112?111201??????????0???1?????????????

Erlikosaurus_andrewsi

?0012???2?0?1?1??1010011100??0001?100000100001000000000??11112100100000020001?

001001001??

0???0000?021?0??00??11??00?0?00?????????????0?

00000010010??00000?000????0?000002000001???10?0100???0000?0?10000??

0000???1?0?0010000?

0010000000??00000000????????0000?0????000?????000?

0?????????0????01?0001100????1?10000?0?00?0?100000000???0??0?00??00?0????000001000000??00?0200?

00000101000?00001?000??00?0?00????????????????00000001101????0???01001000200000000000000??????

0??????????????????????????????????0?00???0000???????00??0???0?

011?????????2???????????????11210?11????????1?0?1?????????????????????????1?01001000????01??????????010?0?

Alxasaurus_elesitaiensis

???210?100????????????1001001??????????0?

01010000?1?0101002?0?1????????????0?000??0?10?0000120?11??0??1?20?21???11?????????1????0?????????

00000?10000??001?1??00?0??00?0?0???00??????????0??????????0??????0????

0????????????????????????????????????0??00????0??00??[01]0?????????????????0?????????0??11??01??????0?0???

010000????????????10?0100???000000???0??0??????????????????????????????1000000?0????????

0?????????????????????

000???

??0??0020??????????????????????0????????????11101?????1??0?

11??0?00??010?0?????01?????????2?????????????????200??0????1?1??0??0????1??1?100???????0000000??1??

0???0?00???11?0????01100??11?1?????1111?????1?00???10?1???0?????????????1????

Neimongosaurus

???21????0?????????????0??001?1??000?110101?

1?0?10?2?1?1?02200????????0001010000??????????????111001?2?????????????????????0??00????0??

000000021?00????0??1??00?0??00?0?????0???????????0???????????????1[12]200?

0?????????????????????????????????????0?????00?0000?0??????????00000200?0?00000?0?11001?0?0?10?0???

010000?0?00?????????????????????0???????????20?00???????0?00000000?0210??????0????????0??????????2?

1100?

000???

??0????2??????????????????????0???????0??1??11???????0??

01?10?0?00???????21?2???????????2?1???1?00??00000?1???0??00????00????00??????100010?2???????00?????

1???0?0111?111001011????1?1121?????111111????10????1??11??0?0??0?100???1?1??

Erliansaurus

???10??

1??????????1???????????????0?0???10?0?0???00000????1?0?1?????????????2??1????10?00?0?0001?0??

00??????????00?????????????????????0?????????????????????0??????

200???0????0??????????????????????????????????01?0?0?10?0???

010000???00010???????100???000000???????????20?00???????0????????????????

0??????????????????????????????????

000???

??

0???????????????????????????????0?0?1?0?01????????????????02?0???1?000??????????????00100???????

0?????????00???100?????00?11????????????????011?111111??11?1???0?1?11111011?0?????????1??????????0?

1??????

Suzhousaurus

??0101??

01000??0??1????0??????????001010000???????????200111001?2020?2?10010020110101100?

00??????????????????0???????1????????00???010????????????????????????0?????0?

0???20010000[01]????????????????20000?00000?01010?1?0?0010?

0???0100000??00?????????????????????00?000?00?1020000?????????????????????0??????????????????????????

2??

??
10111100100000?11010?00??????021020010?0101?00201010????????????????????0???0??01??00?1????1000?0?
2???????00????????????????11?100011????111221211111O??????????????????1?0??01102?0?2???????
Nothronychus
?????1112??11?1?0?0????????????????????????????????00000???????????????????????????1????????????10101?
10?01?1?00?100220?001?????00000?100?00????00000200??10?1?2020221100100201101?1100100?
10001100000021?00???00??????0?0?000?0?010?00????????????????????0????1[12]200???????????
1?????????????????????????????????0010000[01]0??????????00000200?0?00000?0211001?0?0?10?0???
010000???00010???????000???00???00?000000?1020??000000000?00000000?0210??0?000?0????000???????
0???1100?000?
000???
??????????????????????????000?01100???????????????????????????????????????0???????0?0??1010110???0000?
110?00001?0?0?021?20010?1101??02???1?10000?0?000?10?????0?1?0000010?0??1?11?1?0010020000?
00001110?1?2??????1?1110?0001???11112221011111?12101120??0????1?1?0???10?????1???10?
Enigmosaurus
???
??111?01?2020221100??
0201101???????????????????????????????????1????????00???
010???
??00?000000?
10??
2??
???
1??1??????????????????????2?020?10?1101??????????????????????????????????0??01?????
1???1?122121100??
Nanshiungosaurus_brevispinus
???1???????101?
121???
0???

??0?????1???200??

0??

0???

2??

???0?????????1???1??

0????????????????????????21?2??10???01????????????????????????????????????01??0??

1?????????????????????????????????????1??????????????11222??11???

Therizinosaurus

???

?????????????????????????0010?0000????0?00200??????????????????????????????????????0??0?11000000021?

0????00??

00??0???????????????????2000??00000?01?1001?0?0?10?0???

010000?????010???????100???00000??0??

0100???????????????????????????????????

00???

??00?

1010?00100101??001?0????????0??????????10??

0000?????????????????????????1110111111????????????????2?111?0?????????1???01????0?????????

Haplocheirus

?0010???00010?0?12011011101112001?000020100?0?1010??0001?001000001011011?100000110101010??10??

0?0111201??0?00001?0???0?00??????100210001210?0?00010???001?00?101002010010?011000?00000000?

0000?0000000??00?1110001100010001?0???0010?01000000000??0000001?00000?102000000000????00???00???

0???000000??0??0??0000?000?000????0??????????????20000?000000010101??????100???000?00??????????0???

00000??0000000?00?000100200000??????000?0??0???0100?000???0?0?????????00?0?????????0?0000001?

00001??0?0000000?0?01000000?0??11???0??0?01??01?0????000001000000??00??100?00000000000?000????

00??0??0?0??????001????????0?0??0?0000????0???00000??00?00000??00?????????000???????0??1???????????

00?1010100??0100?00?0????????0?01??0???00?0?????0?????00000?00?101000??00?0??010?

110010010001000001110??00??0?101???00000???0000?????????000????????00101???0?1?1?010?????0??0000??1

Alvarezsaurus_calvoi

???01000???
0???0?20?0?2012????????????000?00????????0??1?0000101?00?2??????????????????01?000??0??0??11000000??
00?????????1???0?0??00?0?????0??????????????????????0????
10???00100???0???????????????2?000?????0?0?
0???0??000?00???2????00000000?000???00?02???
0?????????????0????????0??????????
00???
??0???????0001?00????????00?
10?0?0???????????000?000?????????????????0??00?????00????????????0??0??0????????1000???????10?????
101???????1????00?????????00000???????1???0?000?10??????1???0?0??0??0?????????
Patagonykus_puertai
??1?01?
112????012010?2?2??????????????00210???210?????1?0???1????1010?????20????21202??11000000000001210??
0????001??0???????0?????????0????0????????????????????0?????
01??0?00100???????????????????20000?000?00??????0?0?0000??
0?0010000???0??????????1???????????0???0????100200000000000??00?0???????10??
0??????????????????????????????????
0??
???00001?01100?0?????
000?1100??????11???0?0?01?01?1?000?10??1????0?00?????0????00?000?0??1???0??1001?101??1?0?
1111111????????????0?00000??0?????00??0000?00?0?00??1?????????10??1???0???0????1??
Achillesaurus
??
???020???2?????????????????????????????????????10??01?2???????????????????1??????0??1?01000??0???
00?????????1???????????0?????0?????????????????????
0???
??????????????????????????????????????0???????????2????00000000?0??0??
0???
??

??00?0??????????0????????00?0????
0??????????????????????????????11????????????????11?????????????0????????????000???????1???0?
00?????????????0?????0????????????
Mononykus_olecranus
??????00???????112???????????????????????????????????100?????????????????????????2????00???????1?1?
11102001???2?10?2??????1???1000?000200030210?02201100??1????1?201000?2?????3??0??
021010011101121200003000000 1??00???1??0?0??00?0?0???00??????????????????????????
00011???0?0010?0?????210???00?????2?010?0?0?0?010?011?0?
0001000?001000000000??0000???30000???0???0??00???????2000000000000??0000??0?00?0??0??000??????
01?0?????0?0????????
11102??
???0???????1001001000????????
1010100000100?????011???0???01?010000010101?100000100????0??????00??1??0???021?
111001011111111101011???????0100000000000?000?0000??000010000?000?10????????100?10100?0?01???1??
Shuvuuia_deserti
?00101002?01011112011000101 1?01111?1002010000101001001 0???1110?0010100100 2100??0211??000??
0111111011102100?0?2010120121101 00?1000?00020003021??022011000 0101001?201000?2002203??03?
02101001110112120000300000001100000110000001 0000?00??001000000001?010??000000000?000100000000?
101000?10000110?0111000??0[12]0????0100?0010000[01]0?0210?0?00?????20010?000?010101011?0?0001000?
00100000?000?????????30000??000??00?000?00?102000000000000?0000000?00100000?0000020?0001?01?
1010001???????1110200?000?000??0?1??0000?0?00000?00000100???0??0?00??01?0????0?????0??000??00?
0200?000000??0?0?0??00?000?001?0?0???????????????????000000000????????00000?000?0??000000??00??????
0?0???????0001001000101 1?00010101000001 00??0?01110000???01??1??????????0???0????00001????1??00??
1??00110210?110010?1111111??0?01002?0??00??0?0000000?000000000??000?????0000001010 1?0??0?
00110????00?1101?0?
Parvicursor
??
10?????????1??2???1????2?1000???02203??03??2??100011?
11211000030??00?????????1??????????0?00??0??????????????????????0?????

0???0?00?

00??0????????1?

2000000000000??000?000?00?0???????????????0??????????????????

11??

???1000?????

00?????????????????????????0???01??10??00?0?01??000??100??????????00??1????????1?111??????????

11100010????????????0????????????0?????000??0000000010??????????0???1?0????1???1??

Albertonykus

??

??????????????????????????????????211?????1?0???

030?????????

0???

??

??0??

02??

??

????1??????????????????????????????0???00????0???????????????????????????????11???11??

1???0????0???????????1??????????????????

Albinykus

??

??0??????????????????????????????1101??

121100300000????????????????0000?????

0???

???0??????0????????000000000?0000000?00??

0????00????????0??????????????????

11??

??

??????????????????????????????????????0?010000??00???????????00??

1??00000?10??????????0??????????
1??????
Bonapartenykus
??112??
0????????????????????????000210????????????????????????01????????????212?2??1?????????????????????????
0???????????0?0???????0?????????????????????????????????0???
00100???????????????????20000?000?000??10??
100??????????????????????????
0??
???
????????????????0?1000??1?????0?010?010??????????????????0?0?01??????????????????????00?????
00???????????0??????1???011????????????????????????????0??????????????????0?????0????????????????????
10????0?????????
Ceratonykus
?0010???2??10??????????????????11???10?201?0?0?0100??0?????????????01?0??????????????????????????1?
1??????????????????????????10????0?2???0??1????????212?
0003???00????????????????????0?????0???????????????????0???????????????????????00???????1????
111????????????????????????????21?????0??????0000?0???01???????????????????
0???00000000??000?000?00?0?????????????????1????10?
0???????????????0??0??????????????????????????????????0?????????????1?0????0?????0??000??0??02???0?
0000??0?0?0??00?000??0????0???????????????????0??0?00???????????0?0????????????????????????????0???????
0001???0?0??1000???????0??????????????????1?????1?00?
0?????????????1??00?00001???????????01????????????????
Linhenykus
??111?101?1??
1?0???2010020?2???????10?0???1??????????0?21110??????????2??????????????????0?1010001????2?2?0?030?
0000???0????1??????????0?????0???????????????????????????
01???0?0012?0?????210????0?????20??0???0?0?
0???????????????????10000???0??????????31??????00??0?????????1?2000000000000??000?000?001?????0?

0?????????1???????0?????????????

02??

???0???????0001?01000?????0??????

1??????????????????????????0?0??00?0?0???000???00?0??????1??0????????????1011????????1111?1???

0??????????????0????????00???????????01??00?00001???????????0?1?110?0?????????

Xixianykus

???102?

00?21201??0????????????????????????????????????0?01?1??1?200000?20022?3??03?02?010011101121211??3???

000???????11???????00?0?00??0??????????????????????0???

0?0010000??00?000000?1?

2000000000000??000?000?0?????????????????????????????

0??

???1000?

0??????????????????????000110000???01?010??00?0?00010?0??????????????????????0??1???1011????????????

111??11?????????????0??????????0?0000??00001000000000001??????????0????100????1???1??

Nqwebasaurus

?0????????????????02????2010????????0100?00??1?????0?00????????????????????????02????1??????00?

11001???1??????????????????????????0102?000??0?00?10110??????????????????????????00?????0?

0000100010000000?000?1??00??????0?0?0?????0????01?00???00????????????????

10200??????????????????????????????00??????????????????????????0??????????????20????0?0???0101?0??????10?

0????????????????????????00000??00000??????????00????????????00?0????00??0?0??00000????????0???????0?

0??????????0?001?????????????0?0??????0???0??????????0???0??????0?????????????????????????

1??????????????????????000??0??0?0??????????????????????00???

0??????????????????????????000??01?0?1?0?0??????????????????????????0????00?0???00?????0?111?0???1??0??

012?????100??00010000100001???????0??????????????0?????????????????0?000010??????????0???????????1?

1???

Shenzhousaurus_orientalis

???0????????????????21000??10?0??1???000??00?000?0???????????0?00000000??0??0?1?12?0??1????????????

000??1???0???00000???0???????????????????????????010000000??11000100101?000001100??00001?

0??????????????????0??1??1?0011????00???0010??10000010???????00??0?00???0???00?

000????????????????????00?????0??????0?00?0?100?0?0?

0???0?????0?00000?00000?0002?

000??????????????????????00???????????????00000??????????01?00??00000??0?0000?00?0?

0000000000??00???0??0??1???1??10???????

0??????????????????00?00??00?00?0????0????????????????????????000???????00????????????????010100000?

0001?00??0?????????????????01?????0?10??00??1??00?2?0000????????100?0????????00?10????00????????

00000000???00?????????????0???0??0???1????????????1???0

Ornithomimus_edmonticus

?00010?1101101?101021000?0101010110000000000000000001000000?????0000000010?1001?1???????0?001?

1011000011100010100000000100100?????01120012000000?

202001000000110001101011000001100100000110001000100000020??00?011111110121110000?0?

0010001000000001?????0001?000000002100000000?????????0?0?10???000010110??????000000010000[01]0?

0??????????????0??0?0?0?0?01?1001?0?00?000???0000000??00??????1??00000??0000020?000?

000002000000000000?0??0?????0??0??000?1???????0???0??0000000????????00010000?00000?00?0000?01?0?

0100000??0??00???0??0001??01?0????000001000000??01?00????0000101?0000??000000??0????0????????

1??????????1?????????????????00210???0?00000?010????????????010?00?0000100001 1???????????????????10?

00???1?????????????????????????1???????01111001111111?11000???????100??0???00?01????101?0???1??0?

0000?10000000000????0000000000000100?0?????????1???????????10?01

Archaeornithomimus_asiaticus

???00?

101100001110001?100000000?0?????????010200?20000?0110[12]0???000?

0110001101011000001020100000 1100??000?0000020??0000?10??10??1?1??00??0?

001000???0?001000?

[01]0???????????????20?00?00000?0?0??01?0?0010000?00000000000000010000???00000??000??00???

0000000200000000000000?0000??00?0?10?000?????????????0??????0?0????????

00001??

???0???????0??1?00001?00??

00000?00020010?010?010100000?01011?00000000000?000001000????001?101?001?11?0????

0000100000001100001??011??????110?000001100000000000??000000000?000?10?????101?0?1?000?000????
1??
Anserimimus_planinychus
??
?????1?????????????????0??????????1?200?2????00?202001000000011000110101????001?001???????????????
00002???00????11??10??1?1??00?0?
001000??
???0????????0??1?????????????????????????????????????01000??00000?????????????????????????10000??00??
0??0??00?1??????????????????????????????
001???
??
??????10?0??1?1??????????????????????????1?0??????????11011011001??1?0????????100???01?
00??????????????????????000?10?000?????????????????????010?????????????????????????????
Struthiomimus_altus
?00010?110??0??10102100020111 01?110000000000000000010??0001?01??0001000001020001?1?????????001?
1011000011100010100000000100100??????011200120000001201001000000110001101011000001?
0010000011000100010000020??00001111110121110000?0?001000100000001??????00?1?0000??2210????
0???????????0?0?1????000?????0?00???00?0000?0000[01]0?0?????????????200?0?0?000?0101001?0?0010000??
00000000000?100000??00000??0000000?000000000200?00000000000000??00?021000000?10022000000?0??0?
00?00?????????0010000000000?00?0000?01?0?01000000000000???0??0?01??01?0????000010?0000??01?
0000??0000101?00?0??000000??00?0?0??????????????????1000011010?00????00210???0?
000000000????????????00??00?00001000011?????000000000200?110101?10100100?01011?
000000000000010001000001110011111111?1?110000??00001000000110000100011010 0???1??0?
000011000000000000???000?000000000100?0?0?101?1110000?0000?1010?
Gallimimus_bullatus
?00010?1101101010102100020 11?0101100000000000000000010000001?0100000000000102000 1?1?????????
0011101100001110001010000000001001 00??????0112001200000??
2010010000000110001101011000001100100000011000100010000020??00000111111012111 0000?0?
00100010000000 01?????00011000000022 1000?0000000100000 0?0000??00000?0?10200??1000000010000[01]0?

0?????????????20010?0000000101001?0?0010000?0000000000000100001??00000??0000020?

000000000200000000000000000?000?021000000?1002??0000?00??0000000??????00001000000000??0?0000?

01?0?0100000000000???0??0?01??01?0????000001000000??01?0000?00000101000?000000000?000?0?

000???????????????0010000111100000???00210?0?000000000000000???????000000?

00001000011[12]0000000000000020011?010101010100100?01011000000000000000100?10001110111111011001?

110001??000010000001100001000110100???11000000011000000000000??000000000000000010000?

001010111000010000?1010?

Garudimimus_brevipes

?000?????01101????02?00020101000??00000000000000000?00001?0100000000000002?001?1???????0??

0111????0001?10001?100??00?????10???????????????????????????0000001100????????????0??001000001?000?

000?000001000000?01??1110?2?1?0?00?0?0??0001000000000?????00001000000010???00?0000?001000000?

0000??0000010110000??0000000010000???

0??00?

0000000002000000000000000000000?021?0????0000????00???0??001000???????00???000000000002?0000?

00?0?0100000000000???0??0?01??01?0????000001000000??01?0000??000000000000000000000??00?0?000??

00?010???????0010000000010???0???00210???000000000000000????????0?0?00?0000??

0001110000000???????????????010100100?0101?0000000?000000100?10001111??????001000?1?0001??

0000???????????00001011010O??????000??????????000000??0000000000000010000?0010??01??0011??00?111?0

Pelecanimimus_polydon

?00???????1????????2100?2?1??0000??000000?0000??????????????0?000?0000???????000211??0001??000???0?

0?111?00???????????????0??0?00???120????00??0?

2010??1001010????0?????0?????0????10000??0?1?

0????00????0000?0?10???0?0??????????0?0????????????????????00???0?10??????0??????????????0??0?0?0???

0????????????????????????????????????1??00000??00000??0??

000????????????1?????0?00???????????001?00??00010??0?0000?00?000000000000??00???0??0?01??01?00?0?

0000011??000???????????????????????????00??0?000??????????0010000????0??????????????????0??00??

00??????????????????00000000?10???????0?0??????????????????????0?01?????????????????????????????????????

010?1??111??????0????0?2?0?????000001100??????????0?1101??????????0?000????????????????????????1???

0??????1??????????111??1

Harpymimus_okladnikovi

?0?????????????????2100??????0??????000000000000?????????????000000?0000????1?1200??1???0???1???0?

00??10001?100000?00?001????????0?1?0012000000000010?10??00???????1????????????00?00??????0??0?

010000010??000001011????1?0???00?0?0???001??0000??1?0????00??0000??11?10000000???????????

0??????????????0000??00?00000?0000[01]0???????????????????????????10??01?0?0010000?000000000000???

0?010?00000??0000000?0?0000?00?????00000000?00000?00??11??0000?0????????0???????000?????????0?001?

000000000????0????0??0?0?????0000?000???0??0?01??0??????????????????0???1?00??????0???????????

00000??00?0??????????????????????????????????00210??000????????0????????????0???????000??0000?1??

0????0000??2001?00?01?1??0010?????????0??????????00?0??000110010001 1??0??0?11?00????000???

000001000????011???0??01???00000110?000000000???0?00???0?000?100?01??1?1?01??00??00???1????

Beishanlong

??

?????????????????????????010200120000????0100?????????????10???1??00??????000001100?10001000?01??

000?0???1???0??1?1????????01000???????????????????????????

210??0??0???????????????20010?0000000101001?0?0010?

00?0000000000000010????????????????????0??????????200000000000?00000?00????0??0?0?0????????0?

0?????????????????00?

01??

??

0000002001 1?????????????????????00?????0?00000?0???00?????00??1?0???0??1??????????1000000?1??00?0?

011?????????????000100??00????????????0000000001?????????1???10?0??0??0???1??

Sinornithomimus

?0001?????1?0????1?21000??1010001?000000000000000000?000??0???00000000?00????1?1?????????0001?0?

10?0???100?1?1??0?00?0?0?100??????010200120000?011010010000001100011010110000 01?00?

000001100010 00?000?010??0000010111001 21?1??00?0?0010?0100000000 1?????00001000000?

021000000000?????????????00??000001?1?0??????00000001?000?0?0?????????????20010?00?00?0101001?0?

0010000?0000000000??0100001 0?00000??0000000?0000000002000000000000000000??00??1100?0000????????

000???000000????????0?0010000000100?00?0000?01?000100000??0?000???0??0?01??01?0????000??10??000??

01?0000??000??????00000000000??00?0?0?0?????????????????10000????????????00210???0?

000000000????????????0???00?00001?000?????0?0??000000200?100?0101010010 0?0?010000?000?00?0??1?0?

1?00111?1001110?1001?11?00???0000100000011000010 0?11??00???11000000110?000000000???

00000000000001000??0?101?011?000?0?00?101??

Qiupalong

??

??00??1100????????????01100?????????0??0001000002???

00?????????1????????00?0?0???

0??

0???20?0?0?00000?????

00000000?0000?000?02?????????1????????0??????????0????????

00??

??

??????????????10100?00?0001??????????000?010001??0??????????1??110????0????????????????????1??

01??????????????????????????0000??000??0000?00001??????????01??????1???????1??

Kinnareemimus

??

??0???00001???

00????????????????????0?????

0???

0??0?????????0??????

0000000000000?000?02?????????????????????????????????????

00??

??

?????????????????????0????????????????000??00000?00???00???

1????????????????????????????????0???0000???001???????????0??????????????1??

Huaxiagnathus_orientalis

?00?0???????????????0001???1???0?????00??0?????????????????????00?0010????????00010?1010?00??0???

1????????0??????00???0?002010????000000000?00??010000000020?0??000100201?000012?0???0??????0??0?

010000000?0?011??000????00?0??00?0?0010?0100000?0?1?00????0????0000??200???

0000??????????????????????????????????00?00?1??0?[01]0?0????????0000020?00?0?0?0?010100??????10?0???

0???????????????????010????000??00?00??00000??????000?0??00??0??0????100000000????????000????00000?

0?00??000000?01??00000??0?00?0?00?10000000000???1???????????????????????????00?

00?????????????????????????????????????0??02?00???

0?????????????????1?0?00????????0?0??0000??????0??0001100???1?0?000??00000??0?????????????0??????0?0???

0?0?0?00?101000??01?0?00?0?00001000?000??0????????0?????10?????00????0?0?0?0000????0???0????

0000100?????????????????????000???

Sinosauropteryx_prima

000?0???????????????0001???1???0?????0000??????00??0????????????00??01???????1?0001001010?000?01??

100????1?0?0????00?110000201??????00000000?10??010000?00020??????0100201?000?11?0?1?00?0??000?0?

01000000000?011??0000??000?0??00?0?000??010000000?1?00?00?????000?0??20000?000??????????????

0???????0101????????0000?0??0?00[01]0?0???????????????0?00?0?00??010?000?????10?0???00?00????????????

1??0100???0000000?00000??0?2?????00??0??00??00?0???0100000000????????000?10???000?????????0000?

01???0000??1??1????0?1?0?00??0??????0???????????????????????????00??0????????????????????????????????????

0???02?00???0??????????????????000100???????0?0??0010??????0??

00111?0???110?000??00000??0?0???0??0???0??0?00?0???0?000?00?11100???0000?00?0?0?001000?0?0?0??????

010????1100???0000???0?000?00000??000???????0000100?????100??????????????000???

Compsognathus_longipes

?00?0?????????????????00101?1??00???00000????000????000????????0?00000100?1?0010000100101010000?01??

1010???1?0?0???000?0120002010?????0?000000?10????0?000000??????0?0100201000001 2?0??????????00?

0001000000000001100?00????0?0???00?0?001000110000000 1?00000000?0?000??020000?0000??0?0??0??????

0???0000????01?0??0000000010000[01]0?0????????00?0?20000?0?0?0?010100????0?1000???0???

00???????????????010????0?0??00??0?000?00??????00??0?0000?00000?0010000000 0????????00????

000000????????00000?001?001000?1?0100?0001000000000000??0???0??0?000?00?0????0000000?00?0??01?

12????????000???????11?000???????????????????????????0??????????????????02000?00000020000?0????????1?

0000???????000??0000?0????0????????01???1????00????0??0?0?0???0??????0??0000?0???0?000000?101000??

00?0??0?000?0010???0?0????0????10????1100??0000????0???0?00??????00???????0000100?0010100??1?

0????????001???

Juravenator_starki

0000???????????????00010??11000?1???00000?0000?0?????00?????0?0000010???0????000100101??00??0???

1???????0???????00??10000201 0????000000000000????0000000000?01100???????????????????0?????00??????

0?0??000000?10?000?0??0??0??00?0??????010000000?1?00?0?000??0000???20000?000??????????????

0????????????0??????000?00???00??0?0????????[01]00002???0???????0101000?0?0010000??000?000?000???0?

0????100???0000?0???00?000??2??00??????000??0?0000?0010000000 0???????00???00?0?00???00??0?0000?

011000?00?01?0100?00?1?00000000000?00???0??0?00??00?0????0000000??000??01?10????000??00???????11?

000??00?0????????00?????????0??????????????????02000???0?????0???0????????0?

010??????????????????????????0000??00??1?0?000??????0????????0??????0?????0?0???00000?0??101?00??00?

0??0?0???0????0?0?0??0?0?????0????0?0?????000???0?000?0???????0??????????00100?0?????00?1??????0???

00????

Sinocalliopteryx

0000???????????????0001??0???000???00?000?000??0??????????0?0?000001???????1?0001001010100 0?01??

10?????1?0??????00??1000020?0?????0000000??00??0100000003?0?0??000110201?000012?0?????????00???0??

000??0000?011??000?1??0000??00????010?01?000000???00?0000???00000??20000?0000?00?????0????00?????

0????????????00000001000?[01]0?0????????00?0?20000?0?000?010100??????10?0????0??00???????????1??

010????0000000?0000000002??00?0????00?0??00?00??0100000000???????00??10?00000???00??00?000?01???

0?010?0?0000?000??0000000000???0???0??0?00?????0????0????00?00?0??0???2?????00???????????11?0????

0????????????0?????????0???????????????????2?00??00?00????????0?????1???0000??0??000?00010??????0??

000010????1?0?000??00000??0?0???0??????0??0??0?0???0?000?00?100000??01?0?00?0?0?0010?0?0?0????0?

0???0??0?0?00??0??0???00?000?0000????00????????000101?????10???1??????????000???

Mirischia

??

0??0?0???????????????????????????????????????0????????0001?02000???012100100010110????0?????????????

1??????????????????????0010??

0?1?0???0???00?0?

000???

??

???0???000???????????????????????1???????0?0?00?000?1??0?

0???????????????????????0???????0??0????0????0????????????1??????????????????????????????000?0????00??

00???????????????????0???????????0????1??

Ornitholestes_hermanni

?0002???0?0?00?1???0?010?01110001?100101?0??1000001?01011????0000001000?0?000010?00101001?????

011?0101210000??000?0010??1???????????????01?00?????????00001??1?0?0100101000001??0?1???????

0????????00?00????00000?0010?00?0?0000?0?0000?0100000001?01?00001000002?112000000010?0??000000?

0?00???0000?0?0000???0000000010000[01]0???????????????????????????????????0?0?0010000?000000000000??

0??0????1000??0000000?000?00000?0000?????????0000?000?001??00?0????????00??0???0001?0?????????00??

00?1?0000000000000000?000000000??00??0???0000?000?00?0????0000000?0000??01?0000?00?0000100??

00001?000?0001100?00000?01000?????000000000000???0???02000000000020000?00000?00000000???????

0001000002?10?00?0??????10010?01??000000000???0100????000????0?00?0000?0000???????????001??1010?

0000???0???0????0?00??001000000?00???000000???000000??000?????????0?1000?1001?0001????0??0?1010?01

Coelurus_fragilis

???0??

010010000121000?????0?0002?????????????0?0??0?10001????0???????????????????????????01100?000001?0?

0000110?0?0????0?00??00???0???????????0?0????0??????????????????????????

11200??001000??????????????????2?????????????01000?0?

0010000?0000000000000010000???0????????0000?????????0020000000000000??000??????????0000???????????

0??????0???????????

0??

???0???????000010000 2?????0?

000?0??100100?????????????0?0001?000000000?0000?????0?????????????1??00??????????0000??000???0??00?

0100????????110000000000?00?????????00000??00000001?????????0?1?0?11221111??????

Tanycolagreus

?000?????????????????00?0??????0???00110????????0?00????????????????????????10?00??????1?????????????

0011?000?????0????0?0??0???????000000000011?1000000???????????????????????

11001000001100000001000000000000??00???0??000?????0?0???00??????????????????1???1????[01]2000???

0???????????????????00??0?0??????????0?00001000??0?0?????????????20000?000000101000?0?0010000?

0000000000000100000??01000??00000?????????00200000000000000000000?00100?00000????????0?

0?????????????????00000?????001000???????????????????0??????021000000?000?0?????????????????0??0??

00???????0?0?000??00??

0????????????????????????00001????????0011001001000000?????????00???0000000000000000000?000?????

00100000000??0??0?0?100?10000000000000101000?????????????0000000000?????????0?000000000010?????

0??0?100101221011???1??

Tugulusaurus

??

???????0????????????????????????????????0?0?????????????????????????????000?0?100??00110?0?1????

0?????

0???

???

2000000000000???

01??

??

??????00??????????????????000000000000?0????????0?????????0????????????????????????????000001???

1??????????????????????????????????????0?????00??????????????????0001??1????1??

Zuolong

?0000??????????????0000010111?0???000?0000001100000?????1000?????????????????00010?101??1?

010001000????1???000000?0000??????????????????000?0??????0?????00?0100?????????????01100110000110?

00?0???00010?0?00?????0?0100??????00???0???0011000000??0???00??1???0??0002000??0000?????????????

00??0000000??????????010?0?10??0?0??????????????????????????????????0??????10000?

00000000000010000???????????????00?0?0?00?0020000?????????0000?000?0?1???0???????????????0???

000100????????0??00?????0000000000000?00000001000???????1???0??00?0000??????????????????0?????0000?

00000?0?000??10??0010?0001101?00000??0??????????

100???????00000??0???000???0??????00000?????0?00000?0?0?0??0000000000????000?000?00??0????0?????

000??00?0???0????????????000011?????0?0??0000?0?00000??00???0?0???0000000????00??0?????1?0?10??000??

01?0?01??

Bicentenaria

???????????00?1??????0?0??????00??00??????????????00?????????????????????????0?000000001????????????

100?????????0??????????????????????0?00????????????0?1????0??10?????????????????0???00000?10???0?

110???????0??????????1????0?????????????????00??0???0???????????????1???0??????????00????????0??????

00???????????0???0010????0???????????????2???0???0???0?????0?0?001??????00000000?

0??????????????????????0??0???0????2000?00000000?????????????0????????????????????0????????????????

00????????0???????????0???00??0??????????????????????????????0?0000000??????????????00000000?

1??1?2?00??000??0??????

0??????????????????????1?00??0????00????????0??0?????????0000?02???00?0???????00????????0?????

00??????0???00???0???????00???00?????0??????00??00?0??????0??0?????00?????00???????????0?????0?0??0?1?

0?????

Kileskus

???????????????????0?01120111???00000?

101??

???????00?00??0??????????????????????????????1?0000?0???0??00??????1????????0??

001??

???

???1011201???000?0000?0000?

0??

????????????????????100010?????????????

00??00?????00???????????????1??????

0????????????????????????????0???1?????????????????????0?????

Guanlong

?00020?000000?010[01]100011201112000?0011000000100000000000?10????00000010?00??200000001010?100?

000000000111000000000000?0???00???????000000100000?

0100000003000010000100200000000111001000001100?0011000010000000000000000000000000?0?

00100011000000001100?00?11101102?1120000?0010????0000?000?00??0000000??0?0????000000010000?

0???????????????20000?0?????0101?00?0?0010000?000000000000010000???01000??0000000?

000000000200000000000000000000?00100?0000020?20?000?00??0001000??????000000000000112010?1000?

00?1000001010000?003??0000??0??00?10001000001100010??01?1000?00000000000000?1000011?000000?

00000?????????????020?01000100000000000????0?000000010???????010100??????0000100000210000?0?

0000010001000000000000000001000000?0110000000100000?0000000?0001100000100?010010?

1000100000000000010000000000?0010010000000000000000??000000000000010100???1?0011?1001??

1000001??

Sinotyrannus

??????????????????0?01???101?????????????????????????????????00??0?1????????0?0000101?????????????

0??0??00??0?0?

000??0??0?????00?????????1?0000?0??100??0?????

11????????00?001????????????????????????????

0???00?000?
0?0???0???????????00??????01?2?1??10???0??1?00????

1???

????????????0??????????????????????????0???

0001????????????????????????????0?0??????0??????????????0?1????????????????????????????

0??00????

Proceratosaurus_bradleyi

?00?2??????????????00011?01112000?00??????????00?00???????000?00000010?00?

1200000000101011???

??????????????????????????????00??0????0?0??0???????????110000?0?1100?00?11?0?1?2??????

00000100000?????????00???0??00??10?0????

00??

???????????????????????????????????????0???????0?????????????????00?0010112?10?1000?00?1?0000101???

0????????????????0?10001000000110001???????0???0???00000000??0?0????????????0000??000?????0??0?

0??????00100?????0001???10?00000001000?????

11000??0?00????????????

1????0?0?????????????????????????0??0010???1?0?1?????????????????

000???

Dilong_paradoxus

?0002??000000???0?100011??1112000?00110?0000100000000100???????00000000?00??200000001010?1?0000?

0001?0??1000?????0??0?0?0??0???????0000001100?????0000000?00??1??00110201?0?0001?00?000?0??00??

00010000100?0000?00000?0?00000?000?0?0010001000000001100?001111?110201?20?000000????00000???0?

0???000001011000???000??00??00????0?????????????20000?0?0?0?0101000?0?0010000?0000000000000?0??
0????1000??0000000???0?000002000000000000000000??00?0?1000?0000?0?20?00???010000100???????
00000000010002?11?1000100?0?000000000000103??0100?00??000101111000011000 10??00?1100??
00000000010????00000?000100??????????????????020?010?010?0????01001??10?10002?00?11?????
0111000000010000010000 1??????00000110001????10000?0001??000???0110000000000??010000000??
11000????1?01?01001?????00??00???00???00000?00100?00?0000010?0?000000????00??0?000?0001?100???1??
10112????0??0000???
Eotyrannus_lengi
????????????????????000120??12??0???110???????????0?????????0?000?0?0??1???200000001010 12?00?????01?
0??1??????????????????????????000000010????????0?0???0???00000?
0?00???00?0????00?0?????0?????0?1?000?00??10??0121???111?0?211?0000?0???????????????0??00?00100???
00??0???0?0010?0??0???????????????200?0?0?000?0?0?0?0?0?0010000?0000000?0000???????????
00???????????????0??????????????????????????????0???????????????????????1???????????0?00?????1?
00101???????00?0?01????01000?1010??1??0?00?00????????????????0????????????????????01?1??
0??????????????????001??????0?00???????????????????000?1002110100??????????2??00???00???00????
0??????????00??011011?000???????????????????????????01??00?00100?000?0???0??????1?0??0?00????
1000??????0?00????????0000?00???0000100?00??0??????????0000???0?1????0??????011??????1???0?1?1
Juratyrant
??0?00?0000001?
10??00000??0???????????????????????????????????00?00?010000110101100000 11?01???0??10????
0?????????????????????1????????00???0010????????????????????????2????
11???0?00100?0?
0??00?000000000?0?00?000??
00?????????????1??????????????????????????1?
0??
??0????????00?100011?
00100?0?????????????????1000000 11001000?????1100001????????????????????0??01??1??1?????000????????????
0?????????????0000?0???????????000000??000??0000??????????????1??1??????11??0????1??
Xiongguanlong

?0002???00000?????1?000220???0000?001101000?21000000?0001??000??????????????00000?101??

20000000000000111000???0?12?01000??????????????????

0000110??????????????????0??????0?00?????000?????????1?000000???00?0?1121?2102011???0??0000?00?

000??????00???0??000???????????00?

00100??00?0000000??

20000???????0???????????????????????????0???????000100?????????????0010?10001????1?00?01?0?01?

0000100??0010?0100??0??0??0?????0???01000?0???0?0100???001?111110010????100?1?11??00?

100?????????????001001000010?1??????????????????????????????2?000??

0000000011000011????????????????????????111??001???????0111012??????????????00??0???????????1??1?010?

10?????????????00??????000?0??00001???????????000000?????000000???????10????1?????????1???0?0?01??

Dryptosaurus

????????????????????????20?????????????????????????????????????0?0?0??2???????0000101?

1????????????????????????0???00??????????????????10???????10?0????????????[01]?0?0?2???????101?

0100010001000100??2???0??????????0?0????????0?0???????????????00???????????????11????

0????????????????????????????????????0?????????????0?????????????????????????????????0?0?0010000?

00????????????????????????????????0????????002000000000000??000??

0??00?01???????????????????????

0???

??????????????????????????212?1?1???10?????????????????????1?

0???012?1?0111?????1????0??????0?????????????0???????????????000000???????????????00?1???00?????????

00??0000?00001????01??????????0?1??00?0?0??

Appalachiosaurus

????????????????????0?0?201110??????120????????????????????00000?000?0????????00001010?

2???????????????????????0?0?00???000010120?10???10??

010001000100010000002?0?00??????0????0????????0?00100?11000000?1???1?021??????????????000??0??????

000?????0???????????0000???0????????????

0??00????????

002000000000000?0000?000?00????????????????????????????????????00?????1????????000?000?01?010111?

101??1?0020?01?10??0?0??000000010100010?

00??????????????????11?110??1?????????0??1?????2?0?????????????????????????

0??????????????????????????????1?01110???1111111111021?0000????????????0?????0?0???00???????????

0000000???00??0?????0???????????????????00?0??00000001???0???????01?????1??00000??1

Bistahieversor

?1102?????????10?2200002?01110000?001201000?21100000?????????00000000?20??2?000000101012??

0??

0??????????????2???????10??00??00?0??0???????????1200000011?00?00213?0?112??????0000000?????????10??

0????00001???0?0????00??

00???

11??????????0???????????????????0?1011001000?1010101?01011101010101002010001001???10000??

0000002000021100001000??1111111?1?1001011001211111?01000?010011111?????11?111101?10111111100?

11212101100112?10??2?00????111?????????????????0111?1????????????????????????????011?????01101111?

120010000????????????1??1?0?0???????????????????????????0??0??

11001??????1??????????000?11

Albertosaurus_sarcophagus

?1102??000000?10022000022011100000001201000021100000?0001000000000000000020??200000001010120????

0?10?001?100?0????000??0????0???????000000010000???011001031200110000001012001000110??0?00?1000??

0010000020000000100000000000?000?0?0010?01200000011000?00213101112102011000000000???00000100?

00??000001001000 0??0000000010000?0?0????????000002000 0?00000?0001001?0?0010000?

000000000000010??0?????000???00??00?0000000020000000000000000000?00??0100000??020100?000??0??

001100?0110?0000001011101100100000[01]1001[01]1001011101020111002 11[01]1[01]1101?

00100000010001020100200110010001111101111111101011001111111000[01]000111[01]

[01]1111000010110111111110111111100111212111100?12?10?121011???????1?????????????????1111102011111?

102111111021110 1??1??1???11111111112201000000 0??00000101?011010?100010?000??00?000000100?

000000??0?00??110??000000000??0000?000000000101100111101 0111????0100000011

Gorgosaurus_libratus

?11020?000000110022000022011100000000120100002110000010001000000000000000200120000000101 0120?

000001000010100000 0?000000000000?

00001000000001000000101100103120011000000101200100011010010001000?

0001000002000000010000000000000000?0?

001000120000001100010021310111210201100000000000000000100000??0000010?10000??0000000010000[01]0?

0????????0000020000?00000?0001001?0?0010000?00000000000001000010??1000??0000000?

000000000200000000000000000000000?001000000000020100?000100??00110000110?

0000001011101100100000[01]10010100101110102011200211101[02]1?

010001000000100010201002001100100011111011111111101011001111111000100011111111??

001101101111111110111111100111212111100112?10?121011???????1????1101????1?0?

011111020101111102111101021110l?011?1?2?111111111?22010000000100000010110110l0?100010?

0000100000000010000000000000?0000110?000000000??0000?00000000010110011110101l110??0?00000011

Alioramus

?01020?0000001100220 0?0?20111000000012010000211000001000100000000000000200?20??000010101?

000000010000111000000?00?00?0??000??????????????????????????????120011000[01]001012?010??????

001000100??0?01000?02??000??10??000000?0?0000????010??1100000011000l?0213101?12102????000?

00000000000100000??00000100?0000??00000?00100?0?

0??00?0???000??

200000000000000000?0??0?1?0????0020110?00???0??0011000???????0????00111?2????001110111110?

011101010012012100102111010111000100001020000100000010110111201111111100101100111111l?

101110011011101000110110111101101110111100111212111110??10000???000001000011110?

101221011111?????????????????1111010??????1?1111121??00?111?12?0100000??????0???0?1?1?010??

000??????????0????01?0000?000000?0???????????0000??0??00???0?000?1??1101111?1?1??1011??0?000?11

Teratophoneus

?1?0???0???????0??2??????0111?00????1201???????0000??????????00??000?????????000010101??

1??0????????????????????????????????

0??????????????????????????0????0????????????????1?0000?01?????????3?????????0???000????????????10??

0???00?001???0?????0???

1??

100??0????0?01??????????????2?021?0001??10?????????0???01?

1??????0???01??1?1??21?111?1001111011211????????????????????????011?1?120?1????1??1100?11????????

11????????10???????????1?????????????????1?01???1????????????????????011?1?1???????????????

000??????????????????0?0???????????????????????????0??0???

1??????1??????????00??1?

Daspletosaurus

?11020?000000?

1002200002201010000000120100002110000010001000000000000000200120000000101012010000010000 1?100?

0????000??0????0???????000000010000???111001031200110000001012001000110??0100?1000??

00100000200000001000000000000000000?0?0010?0020000001100010121310111210201100000000???00000100?

00???000010010000??000000000010000?0?0????????0000020000?00000?0001001?0?0010000?0000000000000010??

0????1000??000??00?000000000200000000000000000000?00??01000000?020110?000??0??00110000110?

000000101110120011020110021110211110102101201210101[12]111111111111011010200002110010 11[01]1121

1212111111100?111012211111110111001101111[01]100111110111101111111111100111222111101112?

100121011000111011110211111??????111111?211?111?112111??1??111?1??1????2?11????11?12???00000??

11000????1??1?010?1000???000?1000?0??????0?000000000???0011000?0????????????????????????111011110??

1??????????000?11

Tyrannosaurus_rex

?12020?

01100011002200002201010000000120100002110000010001000000000000000200120000000101012010000010 00

01010000001000000000000?0????0000000010000???

11100103120011000000101200100011010010001000100010000020000000100000000000000000?0?

0010000200000021000101213101212102011000000000000000000100000??0000010010000??

0000000010000[01]0?0????????0000020000?0000000001001?0?0010000?0000000000000010000????1000??000??

00?0000000002000000000000000000000000?00100000000020100?000?00??00110000110?

0000001111111200110 2101[01]0[13]0110211110102111 2010??

10121011111011[01]110111102101021100101111 1211[12]121111111001111212301111111111101101111111111111

011020111111111111112112221211011121111121121111110111110211112111111111111102110111111211111102111
1

11112211211111111111122110000000100000010110110 10?1000100000??000000000110000000000010000 1100?

10000000??0000000000000010111011110101111011010000 0011

Tarbosaurus_baatar

??2020?011000?

1002200002201010000000120100002110000010001000000000000002001200000001010120100000100001010000?1000000000000??????000000010000?

01111001031200110000001012001000110100100010001000100000200000001000000000000000?0?

0010000200000021000101213101212102011000000000000000100000??000001001000??000000001000?0?

0?????????????20000?00000?0001001?0?0010000?00000000000010000????1000??0000000?

000000000200000?0000000000000?00100000000020100?000?00??00110000?????

00001111111200110210111301102111101021012010??

1012111111101111101111021010211001011111211212111111100011121230111111111110110111111111111101102

0111111111111121122212?10111211111211211111?111?1021112??1??1?11111102110111?

11211111102111111112211211111111111221100000001000000101101101 0?1000?

0000001000000000110000000000010000110??10000000??0000000000000010111011110101111011??00000011

Merchantville_taxon

??

??

??

??

??1?????0???0???

??

??10?1???????

??0?0????????????????

????????????
