# Supplementary material for: A tyrannosauroid metatarsus from the Merchantville Formation of Delaware increases the diversity of non-tyrannosaurid tyrannosauroids on Appalachia
Source: PeerJ. 2017 Nov 30;5:e4123. doi: 10.7717/peerj.4123 (PMC5712462; doi:10.7717/peerj.4123)
Supplement: Supplemental Information 3 [file peerj-05-4123-s003.tnt]

xread 386 33

Allosaurus

1010002100000000001010000000000001000000100001100000000000000002101110000100100

100010010001???0?000000000010?0??0100?0000000?00000000000000000?

1100000100000000000?0?0100000000000????000?0001000?00100?00000001000?

0000000000000000000000002000000000000000000000000010000010110000002001000[01]

0000100000000001000000000000001001010?000000010001??001000?

0001010001000000010200110000000000001010

Maniraptora

[01]000000000?000000[01]00000000?000000100?00?000011100[01]0000000001000000??1??

00[01]00000?01000?01?00??0?000000000000?0??0010?0010000?

00001000001010010[01]000????0000000000000?0?00??0[01]000??001000?0?0?

0000002[01]1[01]000?1000[01]010000?00000000[01][01]000000000000000001[01]0000000?

0000000[01]0001000000[01]00[01]100001?111100[01]1000?

000000000001000000000000000000?000000000000??100001100??

011000000000100001001000100000000000

Ornithomimosauria

[01]00000000001000000?00000?1?000000000?00?0000100000??00000001?00000??1??

00000000?0210??00?00000?0000001000000?0???010?0000?00??0000000?01000000[01]000??

00000?0000?00000?0??0?00?0??00001000?????0?00002000010?00000000[01]00??????

0011000000???0001000000000000000?0??00000?10?000?00?

00000000000000100[12]000000000000000001000000[01]000[01]010100?010001010[01]00??

000000?0[01]000110011000000000000001000100100?00?00

Compsognathus

0?0001000000000000000000010?000000100?00?0?000000?1?00000000??02000??1???

000000??0010??00?0?????000000000000?10???010?1000??0??000??0?000?0?0??10????

0000??0000?0?000?0????0???????00?????????0???????????????????????????????????0?

000000000[01]?000000???0?????0??0000[01]0[01]0?0??????0?0?0000??00000?000?0?0??

0000010000?0??00100?[01]000000?0[01]0??0010000??0?0000?00?0010000?0?00?00????0?1?

00?000?0????00

Proceratosaurus

000?12000001101112?01000?0?001000100?00?1?00000010???01????0?

0?????????????????????????0?10000?000001010000?1??????0???0???0?0?????

100000000000???0??0?????????????????????0?000??000?????0??0?0?????????????00?

100??????????????0001???110?1?000000001000???0??

111000101???

??

Kileskus

0???12?????110111200100??0?00?000?00?0000?0?000??

000??

???

???????????????0100010???????????????0??

0??

??????????????????????????000?????0

Sinotyrannus

0???12??????10?1?1?1110??0?0??00?????0??1?0?0?????????

1??

???

?????1???????????????????????????0?

01???00?100?0?1?

010001000???

Yutyrannus

10101221?101101111?11002?0?000000100??1?1?0?000010????110??000?011111000??0000??

0210???0?1000???0?011121?01?12100002?0000??1????00?20?00?0?0????????1??????11?

2???10?101?????????0010000??01010?0?11?1?

01020200100???0?0?11?00000?000?00??01?0100???00100?1????1000000000000?0011?000?

00000010??10100110000000??011?0?101001001?01??1???0[01]01010??00?00?0?00

Guanlong

000012?00000101112001000?0?00100?100?00?1000000?10?0?010000000?0012?1?

00110000???010??00?100011000001010000?10???010?1100000?0000001000000000?0?00??

0000000011?0000100000?0??00000?????????????02001?0?010??0000100000?000??0????

0000???10?1?0000000010?????0??101010?010????????00000001000001010000?0?

000000100000010000000000000100000100100000000010100000000000001?

01100000000101000000?00?00

Dilong

000002000001101002?01001?0?001000000100?1?00000000??10000000000001012?1?

00100100??0010??0001000111000010100000?10???000?1110000??000001000000000?100?????

00000000?0000101000?0????????????????????02001?0?0100?0?0010?0?????????????01001??

100?1?010002?00?11???0??10101000100000010000000001000000????????

000000110000001???0??0100001000?0??0?0001110??10??00010??000???011?0000000?

00010000??01?00

Eotyrannus

0???0??????11?100101110?????????????????00?0?0?1???????2?01010000?101001?001??1??0???

000?00????????????????????0??????????????????????????????????0011?1???

0??????????????????????????????001??????0?00??000?

10000?02110100???????????2?2??0?010???00?0?0???00???1?0???????????00???011011011??

0000???00?11??????????????11??101???000?001?00

Juratyrant

0???

??

??

????0????????000?00?10000100?00100?0???????????????????????????????11100001001000111111??

10001100100101??????110000101?????????????

Stokesosaurus

0???

??

??

??110?

0010010001001111????????????????????????????01?????????????

Aviatyrannis

0???

??

??

??100?

0000010000011101?????????0??11????????????????????????????

Timurlengia

00?????????????????00000??????00??00?0100?001?1?

0101??

???????0?????1?010?210011?1?????????????????????????????02?0200?11001000??0????

01000??0111??????????1??121?11?1??11??????2?210??1?0????????????????

00001?????????????????????????????????

10??0

Xiongguanlong

0001000000?1102001????0??0???1?0??00?01?0?001?0?01??1?0001010??001001?001?0100???

000??0??0??????0???0111000??0??0?00?0110000???000?21?111110100?010?1??????100?10?

1111?0??10?0?100????????????001002?00110??000?10?

1??2?2?01?0?0??

00001001000110000010?????????????????????????????????110?101????001111?
0????????????????01110012?0??1???????????????

Dryptosaurus

1???

00???

???

????????????????0?0??1212?1?1????????????????1?

00??10100?????1?????11????????????????????

1??????12?????????1012??1?0011111?????1???

Appalachiosaurus

1??1?0?????????????000001001??000100?01?01001111???1200101?1?1?001101?002?

01010???1?0?

0??

000000010100010?00??????????????????????????????????11?110??01?1??????????0??1?????

2?2?0?0????????????????????????????????0???

1??????12111?1111110???11?1111111110110?0

Bistahieversor

1?0?0011010111200110000010?101000210101?0100111102??

20010101101001100110200001 0?02110??1000000?00?0001210001021100010001000001??10?

311111110??1??10211010021001221111110101??00000?010001110?????1???11?

111111[01]101?1110110011?1?1111100?011001212101100112?1?0?2?2?0100?????

111???????????????????????

0111111??111?1????101?10111111?

110001

Albertosaurus_sarcophagus

1011001101011120011000001001 1[01]1002001[01]100100111021120010101111001110110 21

[01]111100211000100000101010101210101020001 1100?100010??110?

310111110111111211010121001121111101010100100 0111[01][01]1110?

0011012111101111110111111011001????1111100101110121211 1100?12?1?0?21210?

0101??????1???11?????????1?????????0111111021210111111 1?0101121011110110111010000?

1211111012111?021???1???1110111111111112001

Albertosaurus_libratus

101100110101112001?

0000010010[01]100200101001001111021120010101112001110110 2001110?

02110001000[01]0101010100210101020001110001000 10?011003101111101?1?111?

110100210011111111010100001000111111110?

00110121111011111101111111011001111111111001011101212111001 12?100?

2121010101??????1?0111????11101?????1?0??

1111110212101011111101011210111101100110100001121111101 2111?0111?11?2??

11011111111112001

Lythronax

1????0???1?????????00000?0010?00??10?01101101?1102112?011001?1?

0?????????????????????1???001010?02010121000?0??????????????????????????1?11?11?1?

1011?0100211?1?11?111??0?1??????

1?????01?0?1222?211??????1???????1?0??????????????????????????????????

1??????????????????????????????????0??????????121111?11?????????????????10?111?

1??????0??

Alioramus_remotus

000?0????1????????????0??????????1?0??1?0?001????2??2?0111?1???

0???10000?1001?0??11??1?1?3??10?1?01???

1?????01???1001?21111110101????????????????????1101211?1011?11111011?1?1???0?????

01??????0??1?121???????????????????0?

1???

???????????????????????????1?????????

Alioramus_altai

00010011?11????????

000000111111001011111010011110211210111010120111100002101121011110101110001100 1

0101220001010000010011001112011113201111011111101110100210011211111110101??

10111001101110100011012111101111111101101111100111110111100101110121211111 0??

100000????0?100000100010001111 10?11011?21011111??????????????????????????10?

111011?0110100????????????21?111?111111211???00?11111?11??01

Alioramus_remotus_altai

00010011?11????????

0000001111110010[01]1111010011110211210111010120111100002101121011110101110 00110

0101012200010100000100110011120111132 01[01]1110111110111010021001121111110101??

101110011011101000110121111011111110110111110011111011110010111012121111110??
100000????0?100000100010001111 10?11011?21011111??????????????????????????10?

111011?0110100????????????21?111?111111211???00?11111?11??01

Qianzhousaurus

000?00?10111122001100002?1?111101101111101001111021121011101012011110010 2100111?

1111???1?1000?1002?10122000?020000010001001112011113201111101?1?110?1101002100?

12111111010 10????????1????????00110???11101??11111?110??11?0?1????011100010111?

121?11??1011100?00????0?11?01011??1?0?111?102?1011??????111111?

110????????????????????0???11?1100?10??000?????????????????1??1012?1110?0?11111????

0??

Teratophoneus

1?????1??1??????????000010??1??????0?11?01001??102?1???1??????2?011100102?01010??

201010?0???????0???0121?1???201000200?1011???0?1013112111101011?

10111011021111101111 11?1?1?????????????????????1?0121111?11110102011?11?1?0011?1?1??

0100?011?01222?1???111??1???????11000?????????11?11?102????????????111?????1?

0211111011??????????10??1?01101?1??0000?1211111112?1110111211 1211?10?111???????????

Nanuqsaurus

0???

2???

?????????????????????1?011??11?1?21011100??

1???110?10?

10???

??

Daspletosaurus_torosus

1111001101011220011100020001111012002111022011110211210102111120111100112110121

112111111111[01]1110120101210001021110011001O1[01]11211111321211110111111011?

01012111122111111010101101110011001111100 11?1211110111111[01]1011111111111????

111011110110112221111101112?1100212101010100011110111111021111O??????1?1111111?

212111?11111?111112101111011011101?000112111111211111?12210?2?111??1111111?11?1??

Daspletosaurus_horneri

111?00?10101?2?00??10002000111[01]012002111022011110211210102 1111?

01111101120100211121111111111111112??0121000?

021110011001011112111113212111110111101110?

0121101221111110101011011100110011111001101211110111101?101??1?111111????1?

1011110110112221211?11121110?2?210101011 11?????01?11?1021111O???????11???????11?1?

1?????????????10??11????1?1???0?????????111????1?2122111211110111111111111111

Iren_Dabasu_taxon

0???000?1?0?2?010?10????

0????001010????????1??010??????????????????????31???

1110??????????????????????????????????

00??2?

2????

10???

????????????????????????????????????

Zhuchengtyrannus

1??????????????????1010??0?????1??00??110?111?1?

0211???

???

??????1111?01101??????????????????????

11010??

???????????????????????????????????????

Tyrannosaurus_bataar

1111002111112220011101120011101112113011021111110211210102110120 10??1?

1020100211122111110110111012110121101002110002100111111211101321 2111101?

1111011011003121222011111111?

111111011011111111111121111011111 0[01]2011111111011111?11111120110112 2212?

1011121111121211102011111?1111111?10211111??1??1?111111021211101 1111?

11111210111101101110100001121111111211111212211121111011111111112111

Tyrannosaurus_rex

1111002111112220011101121011101112[01]0[13]01102111111021121010211112010??1?

1020100211022111110 11[01]1110121101211011021100021001111112111013[12]121111111111

1011111003121222011111111110111111011011111111111121111011111002011111111101111111

111111201101122212110111211111212111020111111111111111021111012111111111111110212

1110111111111111210111101101110100001121111111211111212211121111011111111112111

YPM_VPPU.021795

??

??

???

??

??????????????????????????????????1??10?10??
